# Rhomboid homologs in mycobacteria: insights from phylogeny and genomic analysis

**DOI:** 10.1186/1471-2180-10-272

**Published:** 2010-10-29

**Authors:** David P Kateete, Moses Okee, Fred A Katabazi, Alfred Okeng, Jeniffer Asiimwe, Henry W Boom, Kathleen D Eisenach, Moses L Joloba

**Affiliations:** 1 Department of Medical Microbiology, School of Biomedical Sciences, Makerere University College of Health Sciences, Kampala, Uganda; 2 Department of Veterinary Parasitology & Microbiology, Faculty of Veterinary Medicine, Makerere University, Kampala, Uganda; 3 Case Western Reserve University, Cleveland, OH, USA; 4 Department of Pathology, University of Arkansas for Medical Sciences, Little Rock, AR, USA

## Abstract

**Background:**

Rhomboids are ubiquitous proteins with diverse functions in all life kingdoms, and are emerging as important factors in the biology of some pathogenic apicomplexa and *Providencia stuartii*. Although prokaryotic genomes contain one rhomboid, actinobacteria can have two or more copies whose sequences have not been analyzed for the presence putative rhomboid catalytic signatures. We report detailed phylogenetic and genomic analyses devoted to prokaryotic rhomboids of an important genus, *Mycobacterium*.

**Results:**

Many mycobacterial genomes contained two phylogenetically distinct active rhomboids orthologous to Rv0110 (rhomboid protease 1) and Rv1337 (rhomboid protease 2) of *Mycobacterium tuberculosis *H37Rv, which were acquired independently. There was a genome-wide conservation and organization of the orthologs of Rv1337 arranged in proximity with glutamate racemase (*mur1*), while the orthologs of Rv0110 appeared evolutionary unstable and were lost in *Mycobacterium leprae *and the *Mycobacterium avium *complex. The orthologs of Rv0110 clustered with eukaryotic rhomboids and contained eukaryotic motifs, suggesting a possible common lineage. A novel nonsense mutation at the Trp73 codon split the rhomboid of *Mycobacterium avium *subsp. *Paratuberculosis *into two hypothetical proteins (MAP2425c and MAP2426c) that are identical to MAV_1554 of *Mycobacterium avium*. Mycobacterial rhomboids contain putative rhomboid catalytic signatures, with the protease active site stabilized by Phenylalanine. The topology and transmembrane helices of the Rv0110 orthologs were similar to those of eukaryotic secretase rhomboids, while those of Rv1337 orthologs were unique. Transcription assays indicated that both mycobacterial rhomboids are possibly expressed.

**Conclusions:**

Mycobacterial rhomboids are active rhomboid proteases with different evolutionary history. The Rv0110 (rhomboid protease 1) orthologs represent prokaryotic rhomboids whose progenitor may be the ancestors of eukaryotic rhomboids. The Rv1337 (rhomboid protease 2) orthologs appear more stable and are conserved nearly in all mycobacteria, possibly alluding to their importance in mycobacteria. MAP2425c and MAP2426c provide the first evidence for a split homologous rhomboid, contrasting whole orthologs of genetically related species. Although valuable insights to the roles of rhomboids are provided, the data herein only lays a foundation for future investigations for the roles of rhomboids in mycobacteria.

## Background

The genus *Mycobacterium *consists of ~148 species [1], of which some are leading human and animal pathogens. Tuberculosis (TB), the most important mycobacterial disease, is caused by genetically related species commonly referred to as "the *Mycobacterium tuberculosis *Complex" (MTC: *Mycobacterium tuberculosis*; *M. bovis*, also the causative agent of bovine TB; *M. bovis *BCG; *M. africanum*; *M. carnetti *and *M. microti *[2]). *M. leprae *and *M. ulcerans *are respectively the causative agents for two other important diseases, Leprosy and Buruli ulcer [3,4]. Besides the three major diseases, *M. avium *subsp. *Paratuberculosis *causes John's disease (a fatal disease of dairy cattle [5]) and is also suspected to cause Crohn's disease in humans [5]. In addition, *M. avium *and other non-tuberculous mycobacteria (NTM) have become important opportunistic pathogens of immunocompromised humans and animals [6,7].

Mycobacteria have versatile lifestyles and habitats, complexities also mirrored by their physiology. While some can be obligate intracellular pathogens (i.e. the MTC species) [8], others are aquatic inhabitants, which can utilize polycyclic aromatic hydrocarbons (i.e. *M. vanbaalenii*) [9]. The biology of pathogenic mycobacteria remains an enigma, despite their importance in human and veterinary medicine. Except for the mycolactone of *M. ulcerans*, glycolipids (such as PDIMs) and proteins (such as ESAT-6) of MTC species [10,11], largely, in contrast to most bacterial pathogens, pathogenic mycobacteria lack obvious virulence factors and the mechanisms in which they cause diseases are still obscure [12]. Genome sequencing projects have provided invaluable tools that are accelerating the understanding of the biology of pathogenic mycobacteria. As such, genome sequencing data has guided the characterization of genes/pathways for microbial pathogens, accelerating discovery of novel control methods for the intractable mycobacterial diseases [5,13-16].

The rhomboid protein family exists in all life kingdoms and has rapidly progressed to represent a ubiquitous family of novel proteins. The knowledge and the universal distribution of rhomboids was engendered and accelerated by functional genomics [17]. The first rhomboid gene was discovered in *Drosophila melanogaster *as a mutation with an abnormally rhomboid-shaped head skeleton [17,18]. Genome sequencing data later revealed that rhomboids occur widely in both eukaryotes and prokaryotes [17]. Many eukaryotic genomes contain several copies of rhomboid-like genes (seven to fifteen) [19], while most bacteria contain one homolog [19].

Despite biochemical similarity in mechanism and specificity, rhomboid proteins function in diverse processes including mitochondrial membrane fusion, apoptosis and stem cell differentiation in eukaryotes [20]. Rhomboid proteases are also involved in life cycles of some apicomplexan parasites, where they participate in red blood cell invasion [21-25]. Rhomboids are now linked to general human diseases such as early-onset blindness, diabetes and pathways of cancerous cells [20,26,27]. In bacteria, aarA of *Providencia stuartii *was the first rhomboid homolog to be characterized, which was shown to mediate a non-canonical type of quorum sensing in this gram negative species [28-30]. Since then, bacterial rhomboids are being characterized, albeit at low rate; gluP of *Bacillus subtilis *is involved in cell division and glucose transport [31], while glpG of *Escherichia coli *[17,32] was the first rhomboid to be crystallized, paving way for delineation of the mechanisms of action for rhomboid proteases [33,34].

Although universally present in all kingdoms, not all rhomboids are active proteases [19,35]. Lemberg and Freeman [35] defined the rhomboid family as genes identified by sequence homology alone, and the rhomboid proteases as a subset that includes only genes with all necessary features for predicted proteolytic activity. As such, rhomboid-like genes in eukaryotic genomes are classified into the active rhomboids, inactive rhomboids (known as the iRhoms) and a diverse group of other proteins related in sequence but predicted to be catalytically inert. The eukaryotic active rhomboids are further divided into two subfamilies: the secretase rhomboids that reside in the secretory pathway or plasma membrane, and the PARL subfamily, which are mitochondrial [35].

Despite their presence in virtually all eubacteria, there is a paucity of information about the functions of bacterial rhomboids. Hitherto, full phylogenetic analysis of rhomboids from the complex and populous prokaryotes has not been done; although it can provide important functional and evolutionary insights [17,35], it is a huge and difficult task to perform at once. Many species of mycobacteria contain two copies of rhomboid homologs whose sequences have not been investigated for the presence of functional signatures. Furthermore, actinobacteria can have up to five copies of rhomboids, the significance of which is currently not known. This study aimed at determining the distribution, evolutionary trends and bioinformatic analysis of rhomboids from an important genus -*Mycobacterium*.

Herein we report that mycobacterial rhomboids are active proteases with different evolutionary history, with Rv0110 orthologs representing a group of prokaryotic rhomboids whose progenitor may be the ancestor for eukaryotic rhomboids.

## Results and discussion

A quest for the role(s) of rhomboids in mycobacteria is overshadowed by their diverse functions across kingdoms and even within species. Their presence across kingdoms implies that rhomboids are unusual useful factors that originated early in the evolution of life and have been conserved [20]. However, neither the reason for their implied significance nor the path of their evolution are understood; the key to answering these questions is rooted in understanding not only the sequence distribution of these genes, but more importantly, their functions across evolution [17,20]. This study reports that mycobacterial rhomboids are active rhomboid-serine-proteases with different evolutionary history. Reverse Transcriptase-PCRs on mycobacterial mRNA indicate that both copies of rhomboids are transcribed.

### The distribution of rhomboids in mycobacteria: a nearly conserved rhomboid with unique genome organization across the genus

**Figure 1:**
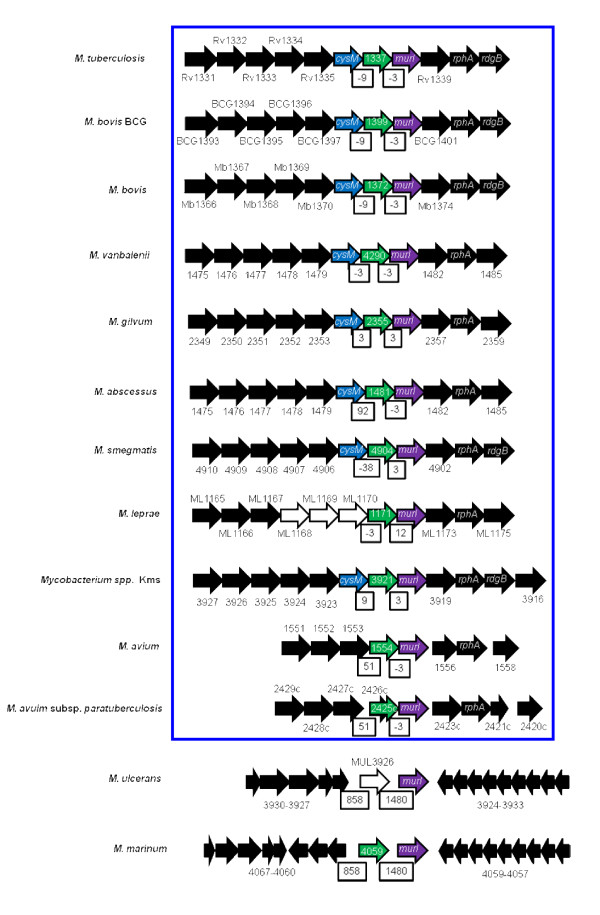
**Genomic arrangement for Rv1337 mycobacterial orthologs**. Unique genome organization occurs for Rv1337 orthologs across the genus. *mur1 *was downstream and *cysM *upstream of the rhomboids (except *M. marinum *and MAC species). Colored block arrows: blue, *cysM*; green, rhomboid homologs; purple, *mur1*; black, rhomboid surrounding genes; white, pseudogene. White boxes indicate distances between rhomboids and upstream and downstream genes. Boxed (blue) are the species with similar arrangement for the rhomboids.

In determining the distribution of rhomboid homologs in mycobacteria, we used the two rhomboids of *M. tuberculosis *H37Rv, Rv0110 (rhomboid protease 1) and Rv1337 (rhomboid protease 2) as reference and query sequences. Many mycobacterial genomes contained two rhomboids, which were orthologous either to Rv0110 or Rv1337. However, there was only one homolog in the genomes of the MAC (*Mycobacterium avium *complex) species, *M. leprae *and *M. ulcerans*, which were orthologous either to Rv1337 (MAC and *M. leprae *rhomboids) or Rv0110 (*M. ulcerans *rhomboid). *M. ulcerans *was the only mycobacterial species with an ortholog of Rv0110 as a sole rhomboid. Thus, with the exception of *M. ulcerans *which had a rhomboid-like element (MUL_3926, pseudogene), there is a genome-wide conservation of the rhomboids orthologous to Rv1337 (rhomboid protease 2) in mycobacteria (figure 1).

Despite evolutionary differences across the genus, the Rv1337 mycobacterial orthologs shared a unique genome organization at the rhomboid locus, with many of the rhomboid surrounding genes conserved (figure 1). Typically, upstream and downstream of the rhomboid were *cysM *(cysteine synthetase) and *mur1 *(glutamate racemase) encoding genes. Since Rv1337 orthologs are almost inseparable from *mur1 *and *cysM*, it is likely that they are co-transcribed (polycistronic) or functional partners. As such, we may consider the cluster containing mycobacterial Rv1337 orthologs as a putative operon. According to Sassetti et al [36,37], many of the rhomboid surrounding genes are essential while others (including rhomboid protease 2, Rv1337) are required for the survival of the tubercle bacillus in macrophages [38].

Despite massive gene decay in *M. leprae*, ML1171 rhomboid had similar genome arrangement observed for mycobacterial species. Upstream of ML1171 were gene elements (pseudogenes) ML1168, ML1169 and ML1170 (the homolog of *cysM *which is conserved downstream most Rv1337 orthologs). Similar to *M. lepare*, the MAC species also had an ortholog of Rv1337 as a sole rhomboid; perhaps the ortholog of Rv0110 was lost in the progenitor for MAC and *M. leprae *(these species are phylogenetically related and appear more ancient in comparison to *M. marinum, M. ulcerans *and MTC species [39]). In contrast to most mycobacterial genomes, *cysM *was further upstream the *M. marinum *rhomboid (MMAR_4059); and despite being genetically related to MTC species [40], MMAR_ 4059 does not share much of the genome organization observed for Rv1337 MTC orthologs (figure 1).

The rhomboid-like element of *M. ulcerans *(MUL_3926, pseudogene) was identical to MMAR_4059 (~96% similarity to MMAR_4059) with a 42 bp insertion at the beginning and eight single nucleotide polymorphisms (SNPs). Perhaps the insertion disrupted the open reading frame (ORF) of MUL_3926, converting it into a pseudogene. Interestingly, MUL_3926 nearly assumed the unique organization observed for mycobacterial orthologs of Rv1337, in which the rhomboid element was upstream of *mur1*.

The functional and evolutionary significance for the unique organization of the Rv1337 orthologs in mycobacteria is not clear. Since physiological roles are not yet ascribed to mycobacterial rhomboids, it is not certain whether MUL_3926 (psuedogene) would mimic similar roles in that it almost assumed similar genomic organization (note: functions have been ascribed to certain pseudogenes [41-43]). However, the fact that *M. ulcerans *is a new species (recently evolved from *M. marinum *[40]) that has undergone reductive evolution, MUL_3926 could be a consequence of these recent phenomena [44]. Interestingly, MUL_3926 was the only rhomboid-like element in mycobacteria.

**Figure 2:**
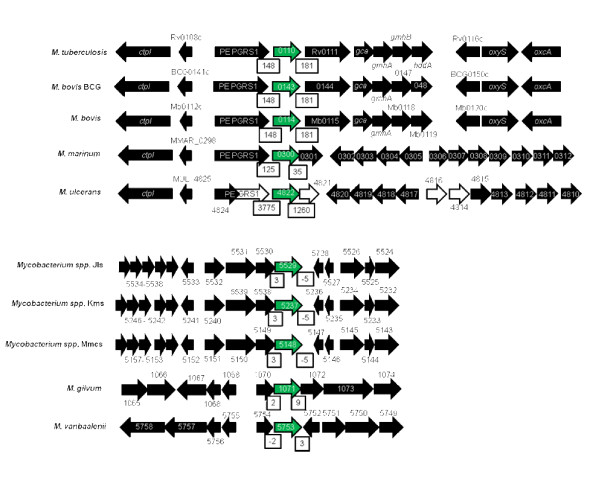
**The genome organization for Rv0110 mycobacterial orthologs not conserved**. White open arrows indicate pseudogenes; green solid arrows, Rv0110 orthologs; black solid arrows, rhomboid surrounding genes; open boxes, distances between rhomboids and neighboring genes (which were big except in *M. gilvum*, *M. vanbaalenii*, and *Mycobacterium *spp. JLS, Mks and Mmcs).

In contrast, the genome organization for Rv0110 orthologs was not conserved, and mirrored the genetic relatedness of mycobacteria (figure 2). As such, the orthologs from MTC species, *M. marinum *and *M. ulcerans*, which are genetically related and are assumed to have the same *M. marinum*-like progenitor [39,40,45,46] had similar organization for Rv0110 ortholog. Downstream and upstream of the rhomboid were respectively, the transmembrane acyltransferase and the Proline-Glutamate polymorphic GC rich-repetitive sequence (*PE-PGRS*) encoding genes. *PE-PGRS *occurs widely in *M. marinum *and MTC genomes [39] but it was a pseudogene upstream MUL_4822 of *M. ulcerans*. The distances between MTC Rv0110 orthologs and the neighboring genes were long, in contrast to the short distances between Rv1337 rhomboids and their neighboring genes.

Similarly, the genome organization for the Rv0110 orthologs of *M. gilvum*, *M. vanbaalenii *and *Mycobacterium *species M.Jls, Mkms and Mmcs was also similar. Upstream and downstream the rhomboid was, respectively, the glyoxalase/bleomycin resistance protein/dioxygenase encoding gene and a gene that encodes a hypothetical protein. In contrast to MTC species, the Rv0110 orthologs in these species were close or contiguous with the neighboring genes (figure 2). The genome organization of MAB_0026 of *M. abscessus *and MSMEG_5036 of *M. smegmatis *were unique to these species (not shown).

Many bacterial genomes contain a single copy of rhomboid. However, filamentous actinobacteria such as *Streptomyces coelator *and *Streptomyces scabiei *have as many as four or five copies of rhomboid-like genes. Since multi-copy rhomboids in prokaryotic genomes are not yet characterized, it is not certain whether prokaryotic rhomboids can also have diverse functions, similar to multi-copy rhomboids in eukaryotic genomes. Mycobacteria and actinobacteria at large exhibit diverse physiological and metabolic properties. It remains to be determined whether the diversity in number, nature and functions of rhomboids can contribute to the complex lifestyles of these organisms [8].

### Similarity between the two mycobacterial rhomboid paralogs

Across the genus, the similarity between the two mycobacterial rhomboid paralogs was as low as that between prokaryotic and eukaryotic rhomboids (~10-20% identity) [19]. Since paralogs perform biologically distinct functions [47], the two mycobacterial rhomboids may have distinct roles. Eukaryotic rhomboid paralogs are also dissimilar and differ in functions in a particular species [17]. In contrast, the orthologs had significantly high homology (see table 1), with an average identity of 74%. Rv0110 orthologs within the MTC and MAC species had an identity of ~100% while those from other mycobacterial species had identities ranging from 61 to 78% (table 1). The exception was MAB_0026 of *M. abscessus*, which shared a significantly low homology with Rv0110 (38% identity at 214 amino acid overlap). This could be due to the large evolutionary distance between *M. abscessus *and other mycobacteria. Since proteins of ~70% identity or more are likely to have similar functions [48], MAB_0026 may have unique roles.

**Table 1:** The distribution and similarity^a ^of mycobacterial rhomboids

	*Orthologs of Rv0110 *(rhomboid protease 1)					
			**Query: Rv0110**		**Query: Rv1337**	

**Species/strain**	**Rhomboid**	**Length**	**%Identity**	**E-value**	**%Identity**	**E-value**

**^b^**H37Rv	Rv0110	284	**100**	5e-143	26	3e-06

**^c^**BCG Tokyo	JTY_0114	284	**100**	3e-143	26	3e-06

*M. bovis*	Mb0114	284	**100**	3e-143	26	3e-06

*M.ulcerans***^†^**	MUL_4822	254	**78**	5e-104	27	1e-04

*M. marinum*	MMAR_0300	289	**77**	1e-103	26	2e-06

**^d^**M.sp. JLS	Mjls_5529	289	**67**	7e-97	NS	5e-06

**^e^**M.sp. Kms	Mkms_5237	289	**66**	2e-96	NS	3e-06

*M. smegmatis*	MSMEG_5036	250	**64**	8e-90	NS	7e-09

*M. vanbaalenii*	Mvan_5753	290	**61**	6e-77	NS	6e-08

*M. gilvum*	Mflv_1071	279	**61**	7e-73	NS	2e-06

*M. abscessus*	MAB_0026	287	**38**	7e-38	NS	1e-04

	***Orthologs of Rv1337 *(rhomboid protease 2)**					

H37Rv	Rv1337	240	27	7e-06	**100**	7e-137

BCG Tokyo	JTY_1373	240	27	7e-06	**100**	7e-137

*M. bovis*	Mb1372	240	27	7e-06	**100**	7e-137

*M. marinum*	MMAR_4059	222	26	8e-07	**83**	2e-106

*M. avium***^†^**	MAV_1554	223	28	9e-05	**75**	7e-95

*M. leprae***^†^**	ML1171	238	27	1e-04	**73**	7e-94

**^f^**MAP**^†^**	MAP2425c	223	NS	1e-04	**74**	6e-91

*M. smegmatis*	MSMEG_4904	219	NS	1e-05	**73**	9e-89

M.sp. JLS	Mjls_3833	229	26	1e-04	**67**	7e-81

M.sp. Kms	Mkms_3921	229	26	1e-04	**67**	7e-81

*M. vanbaalenii*	Mvan_4290	225	NS	4e-05	**67**	9e-77

*M. gilvum*	Mflv_2355	225	27	7e-04	**66**	9e-68

*M. abscessus*	MAB_1481	225	NS	8e-05	**61**	4e-67

**^a^**: In comparison to Rv0110 and Rv1337 of *M. tuberculosis *H37Rv; lengths refer to number of amino acids

**^b^**: *Mycobacterium tuberculosis*

**^c^**: *Mycobacterium bovis*

**^d^**: *Mycobacterium *species Jls

**^e^**: *Mycobacterium *species Kms

**^f^**: *Mycobacterium avium *subspecies *Paratuberculosis*

**^†^: **Species with one rhomboid

NS: Not Significant (< 10% identity).

### The two mycobacterial rhomboids were acquired independently

**Figure 3:**
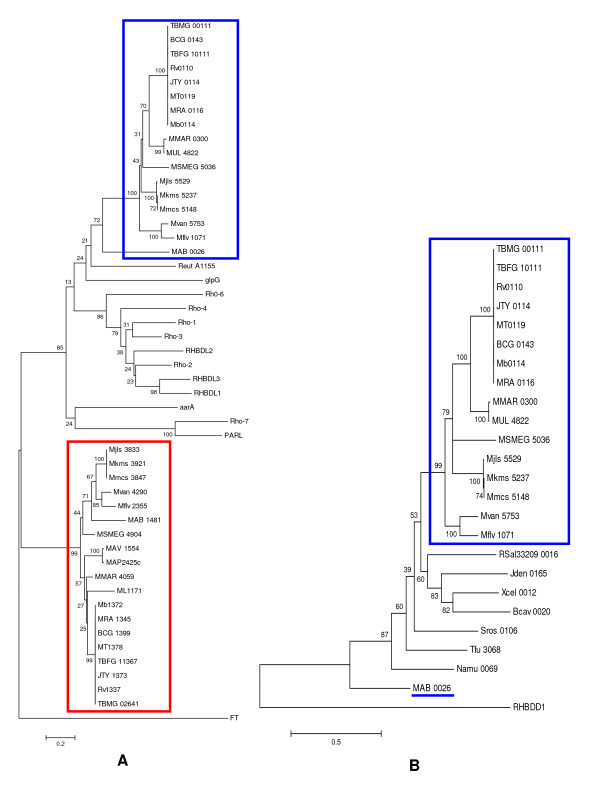
**Mycobacterial rhomboids have different evolutionary history**. **A: **Mycobacterial rhomboids clustered into two distinct clades (boxed blue and red). The Rv0110 mycobacterial orthologs (boxed blue) clustered with eukaryotic active rhomboids (unboxed). The Rv1337 mycobacterial orthologs (boxed red) appeared unique. Mycobacterial rhomboids could have been acquired at the same time, and the orthologs of Rv0110 were eventually lost in the MAC species and *M. leprae*. Mouse-protein farnesyl transferase, FT, [GenBank: AAI38303] was the outgroup. **B: **MAB0026 of *M. abscessus *(underlined blue) is conspicuously distant from its mycobacterial orthologs (boxed blue).

To determine evolutionary relationship between the two rhomboid paralogs, phylogenetic analysis was done and included distant eukaryotic and prokaryotic rhomboids. The mycobacterial rhomboids clustered into two distinct clades with high Bootsrap values (99-100%), indicating that the rhomboids could have been acquired independently (figure 3A). Each clade consisted of rhomboids orthologous either to Rv0110 or Rv1337, grouped according to genetic relatedness of mycobacteria [39], with MAB_0026 of *M. abscessus *appearing the most distant. The phylogenetic analysis confirmed that the two mycobacterial rhomboids are paralogs, but their progenitor could not be determined. Thus, the mycobacterial rhomboid paralogs may be "outparalogs" (i.e. they could have resulted from duplication(s) preceding a speciation event [47]), while the orthologs could have originated from a single ancestral gene in the last common ancestor [47]). The Neighbor-Joining and Minimum Evolution phylogenetic trees were compared and gave almost comparable results.

The Rv0110 (rhomboid protease 1) mycobacterial orthologs (boxed blue) clustered with eukaryotic secretase and PARL rhomboids with a high Bootstrap value (85%, figure 3A). When grouped with eukaryotic iRhoms, the Bootstrap value for this clade increased to 90%, with iRhoms forming a distinct clade (not shown). The Rv0110 mycobacterial orthologs may represent prokaryotic rhomboids with similar lineage or progenitor for eukaryotic active rhomboids. This was previously noted by Koonin et al [19], who hinted on a subfamily of eukaryotic rhomboids that clustered with rhomboids of Gram positive bacteria. Indeed, the Rv0110 mycobacterial orthologs contained extra eukaryotic motifs and have topologies similar to that of rho-1 of drosophila. Koonin et al [19] alluded that rhomboids could have emerged in a bacterial lineage and were eventually widely disseminated (to other life kingdoms) by horizontal transfer [19]. Conversely, the Rv1337 mycobacterial orthologs (boxed red) formed a distinct clade, different from Rv0110 mycabacterial orthologs. These rhomboids appeared evolutionary stable and did not cluster with eukaryotic rhomboids.

MAB_0026 of *M. abscess *which had low homology with Rv0110 also appeared distant and clustered poorly with mycobacterial orthologs, in contrast with its paralog MAB_1481 (figure 3A). Since orthologs have an ancestral gene in the last common ancestor [47], MAB_0026 could be a "pseudoortholog" (i.e. it is a distant paralog that appears orthologous due to differential, lineage-specific gene loss [47]). In phylogenetic analysis of mycobacterial rhomboids orthologous to Rv0110, MAB_0026 was also distant from rhomboids of other actinobacteria (figure 3B). Since *M. abscessus *is one of the earliest species to diverge of all mycobacterial species [39], the low homology could reflect evolutionary distance or stability of this rhomboid. However, the high homology of MAB_1481 (62% identity with Rv1337) contrasts the low homology of MAB_0026 (38% identity with Rv0110), negating the notion of evolutionary distance and instead favors evolutionary stability of MAB_0026.

### Mycobacterial rhomboids are active rhomboid-serine-proteases

**Figure 4:**
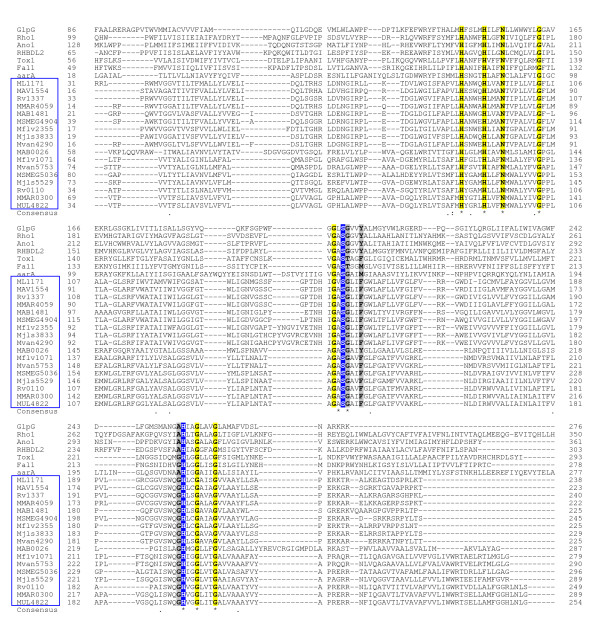
**Mycobacterial rhomboids are active rhomboid proteases**. Highlighted in blue are the rhomboid catalytic dyad residues (Ser201 and His254); yellow, the invariant residues in this alignment; grey, the rhomboid family invariant residues that were not conserved in this alignment. Locus tags for mycobaterial rhomboids are boxed blue. Included: aarA [GenBank: L28755] of *P. stuartii*; glpG [GenBank: AAA23890] of *E. coli*; rho-1 [GenBank: AAF47496.1] of *Drosophila*; (Ano1) AgaP_AGAP004737 [GenBank: XP_318085] of *Anopheles gambiensi*; (Tox1) [GenBank: #Q695U0] of *Toxoplasma gondii*; (Fal1) PF11_0149 [GenBank: XP_001347820] of *P. falciparum *and RHBDL2 [GenBank: NP_060291.2] of human.

Multiple sequence alignment revealed that all mycobacteria rhomboids contain the putative rhomboid catalytic residues Gly199, Ser201 and His254. The mycobacterial rhomboids also contained two additional C-terminal Histidins (His145 and His150, which together with His254 are universally conserved in the rhomboid proteins [19]) and five invariant transmembrane residues (Gly202, Gly257, Gly261, Asn154 and Ala200) that are also conserved in many rhomboid proteins [33]. However in mycobacteria, Ala252 which occurs in many eukaryotic and prokaryotic rhomboids was substituted by Gly (figure 4). Furthermore, Tyr205 which stabilizes the rhomboid protease active site of glpG [17,33] and of many rhomboid proteases was only conserved in MAB_0026 of *M. abscessus*, being substituted by Phe in mycobacterial rhomboids (figure 4). Thus, Phe is the stabilizing residue in the protease active site for majority of mycobacterial rhomboids (Phe is an additional stabilizing residue for rhomboid proteases [17]).

**Figure 5:**
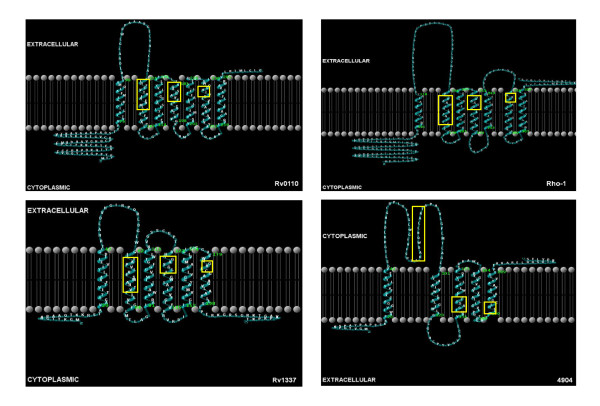
**The topology of mycobacterial rhomboids**. Boxed (yellow) are the transmembrane domains containing the rhomboid catalytic residues and locations for the C-termini conserved residues. The Rv0110 mycobacterial orthologs formed topologies similar to those of the secretase eukaryotic rhomboid rho-1. The Rv1337 mycobacterial orthologs formed either six or five TMHs. The orthologs of pathogenic mycobcateria formed six TMHs while the orthologs of non-pathogenic mycobacteria formed five TMHs.

The nature of the transmembrane helices (TMHs) formed by mycobacterial rhomboids was analyzed to determine whether they conform to those of active rhomboid proteases. Mycobacterial orthologs of Rv0110 formed seven TMHs and topologies similar to those of eukaryotic rhomboid rho-1 of *Drosophila *(see figure 5). As in rho-1, the rhomboid catalytic residues GxSx & H (Gly199, Ser201 and His254, × being any residue) were localized respectively, in TMH4 and TMH6 (see figure 5 and details in additional file 1). In mycobacterial orthologs of Rv0110, the two C-terminal histidine and asparagine (His145, His150 and Asn154) were localized in TMH2, in contrast to eukaryotic rhomboid proteases which have these residues in TMH3 [17,19,23]. However, in our analyses, we found His145, His150 and Asn154 in TMH2 in rho-1, similar to Rv0110 (see additional file 2). Despite the proteins being evolutionary diverse, other studies found the overall structure of TMHs of rhomboid proteases conserved, with eukaryotic rhomboid proteases containing seven TMHs while archaea and eubacteria contain six [23,49]. It is not clear whether these similarities infer evolutionary or functional significance; similar topologies with eukaryotic rhomboids could imply occurrence of a common bacterial universal progenitor for the eukaryotic rhomboids [19]. Nevertheless, prokaryotic and eukaryotic integral transmembrane proteins can have similar architecture, with striking similarity in the amino acid frequency distribution in their TMHs [50].

In contrast, the mycobacterial orthologs of Rv1337 formed either six or five TMHs, as observed in most bacterial and archaeal rhomboids [19]. The orthologs of pathogenic mycobacteria formed six TMHs, while those of non-pathogenic mycobacteria formed five (see figure 5). The GxSx and H catalytic residues were found respectively, either in TMH4 and TMH6 (for Rv1337 orthologs of pathogenic mycobacterial with six TMHs -see details in additional file 3) or in TMH3 and TMH5 (for Rv1337 orthologs of non pathogenic mycobacterial with five TMHs, see additional file 4). The mycobacterial orthologs with six TMHs had the two C-terminal His and Asn residues in TMH2, as in the Rv0110 orthologs; however, in the orthologs with five TMHs, these residues were outside the TMHs (see additional file 4). Although His145, His150 and Asn154 are not essential for catalytic activity [33], it is not clear whether their absence in TMHs can affect functionality. This seems unlikely in that functions have been ascribed to the catalytically inert eukaryotic iRhoms lacking the minimum catalytic sites [26,27]. Alternatively, the observed differences may imply functional divergence, with rhomboids of pathogenic mycobacteria being functionally different from those of non-pathogenic mycobacteria. Indeed, Rv1337 was essential for the survival of the tubercle bacilli in macrophages [38]. Nevertheless, experimental evidence will be necessary for validation of these assertions.

### Extra protein domains in mycobacterial rhomboids

Mycobacterial rhomboids contained extra protein motifs, many of which were eukaryotic. The orthologs of Rv0110 contained diverse eukaryotic motifs, while the Rv1337 orthologs maintained a fairly constant number and type of motifs, either fungal cellulose binding domain or bacterial putative redox-active protein domains (table 2). It is difficult to account for the origin of eukaryotic motifs in mycobacterial rhomboids; nevertheless, extra protein motifs are common in eukaryotic rhomboids where their significance is also not known [17]. Since eukaryotic rhomboids are presumed to have been acquired from bacteria through horizontal gene transfer mechanisms [19], the extra protein motifs may have originated from prokaryotic progenitors. Mycobacterial rhomboids also contained N-signal peptides and eukaryotic subcellular localization target signals which were either mitochondrial or secretory (see table 2), with scores higher than or comparable to those of rho-7 and PARL. These observations further allude to a common ancestor for mycobacterial and eukaryotic active rhomboids [17].

**Table 2:** Extra protein motifs in mycobacterial rhomboids

Species/strain	Rhomboid	Number of ^a^TMHs	TMH with active Site	Extra motif	E-value	Target signal
**^b^**H37Rv	Rv0110	7	4 & 6	DUF1751**^1^**	0.27	Mitochondrial

				Siva**^2^**	0.68	

				Zf-B_box**^3^**	0.00021	

*M. marinum*	MMAR_0300	7	4 & 6	Zf-B_box	0.00012	Other

				FixQ**^4^**	0.016	

*M. ulcerans*	MUL_4822	7	4 & 6	EcsB**^5^**	0.17	Mitochondrial

**^c^**M. sp Jls	Mjls_5528	7	4 & 6	IBR**^6^**	0.301	Other

				Zf-B_box	0.013	

				Dynactin p62**^7^**	0.24	

				Tim17**^8^**	0.36	

*M. vanbaalenii*	Mvan_5753	7	4 & 6	Zf-B_box	0.0044	Other

				Dynactin p62	0.11	

				DUF1751	0.028	

*M. gilvum*	Mflv_1071	7	4 & 6	Zf-B_box	0.015	Other

				DUF1751	0.02	

*M. smegmatis*	MSMEG_5036	7	4 & 6	-		Mitochondrial

*M. abscessus*	MAB_0026	7	4 & 6	Zf-B_box	0.0064	Other

H37Rv	Rv1337	6	4 & 6	CBM_1**^9^**	0.17	Mitochondrial

*M. marinum*	MMAR_4059	6	4 & 6	C_GCAxxG_C_C**^10^**	0.0062	Secretory

*M. avium*	MAV_1554	6	4 & 6	C_GCAxxG_C_C	0.0099	Secretory

*M. leprae*	ML1171	6	4 & 6	C_GCAxxG_C_C	0.031	Other

*M. abscessus*	MAB_1481	6	4 & 6	-		Other

*M. smegamatis*	MSMEG_4904	5	3 & 5	C_GCAxxG_C_C	0.025	Secretory

M. sp Jls	Mjls_3833	5	3 & 5	DUF2154**^11^**	0.6	Secretory

*M. vanbaalenii*	Mvan_4290	5	3 & 5	-		Secretory

*M. gilvum*	Mflv_2355	5	3 & 5	-		Secretory

The rhomboid family domain was excluded

-: Extra domain not detected

Other: cellular localization target other than secretory and mitochondrial

^a^: Transmembrane helices

^b^: *Mycobacterium tuberculosis*

**^c^**: *Mycobacterium *species Jls

**^1^**: Eukaryotic integral membrane protein

**^2^**: Cd27 binding protein

**^3^**: B-box zinc finger

**^4^**:Cbb3-type cytochrome oxidase component

**^5^**: Bacterial ABC transporter protein

**^6^**: In Between Ring 'IBR' fingers

**^7^**: Dynactin p62 family

**^8^**: Tim17/Tim22/Tim23 family

**^9^**: Fungal cellulose binding domain

**^10^**: Putative redox-active protein

**^11^**: Predicted membrane protein

### A novel nonsense mutation at the Trp73 codon split the MAP rhomboid into two hypothetical proteins

**Figure 6:**
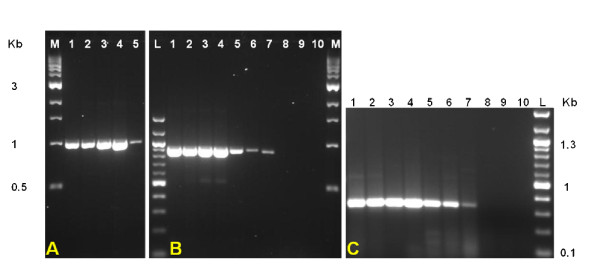
**Transcription analysis of mycobacterial rhomboids**. A. RT-PCR amplification of Rv0110 cDNA from MTC and *M. smegmatis *mRNA. Lanes: M, 1 kbp DNA ladder; 1, *M. tuberculosis *H37Rv; 2, *M. tuberculosis *BN44; 3, *M. bovis *BCG; 4, *M. bovis *JN55 and 5, *M. smegmatis *SMR5. B. RT-PCR amplification of Rv1337 cDNA from MTC, MAC and *M. smegmatis *mRNA. Lanes: L, 100 bp DNA ladder; 1, *M. tuberculosis *H37Rv; 2, *M. tuberculosis *BN44; 3, *M. bovis *BCG; 4, *M. bovis *JN55; 5, *M. avium*; 6, *M. avium *subsp. *Paratuberculosis*; 7, *M. smegmatis *SMR5; 8, negative control (*M. tuberculosis *mRNA, not reverse transcribed); 9, negative control (*E. coli *mRNA, reverse transcribed); 10, negative control (water). C: Similar assays as in B showing cDNA amplification (~350 bp) of the internal fragment of Rv1337 othologs. Negative controls for panel "A" (not shown) were similar to 8, 9 & 10.

The annotated rhomboid of *M. avium *subsp. *Paratuberculosis *(MAP) in the genome databases appeared truncated; MAP_2425c (hypothetical protein) was significantly shorter than MAV_1554 of genetically related *M. avium *(147 vs. 223 residues, respectively). Upstream of MAP_2425c was MAP_2426c (74 residues), similar to the amino-terminal portion of MAV_1554 (100% identity) while the former (MAP_2425c) was similar to the carboxyl-terminal portion of MAV_1554 (100% identity). MAP_2425c and MAP_2426c were separated by 10 bp that translate into three residues (Gln, His and Lys, present in similar location in MAV_1554) and a stop codon TGA, at nucleotide position 217, which split the homolog into two ORFs. Because MAP and *M. avium *are genetically related, initially, we thought MAP2425c and MAP2426c are truncated portions (resulting from genome annotation errors) and should have been a whole rhomboid of MAP. Thus, we aimed to determine the correct annotation for the MAP rhomboid. Using MAV_1554 specific primers, we PCR-amplified and sequenced homologs of MAP2425c and MAP2426c (954 bp) from a cattle isolate of MAP (strain 27, see table 3); the amplicon was similar to MAP2426c and MAP2425c (containing an internal stop codon TGA at nucleotide positions 217-219, and 10 bp translating into residues Gln, His and Lys, in similar location as those of MAV1554). Thus, we confirmed the annotations for MAP2425c (hypothetical protein) and MAP2426c (hypothetical protein). It was later revealed that a nonsense mutation at nucleotide positions 217-219 (formerly TGG, the codon for Trp73), substituted guanine at the wobble position for adenine, creating a stop codon (i.e. TGG[Trp73]→TGA[stop codon]). Usually, nonsense mutations disrupt ORFs resulting in truncated and non-functional proteins; however, this rare scenario resulted into two unique ORFs of MAP, providing the first evidence of a split rhomboid, contrasting whole orthologs of genetically related species. Although the significance of this is currently not known, cDNA was amplified from both ORFs, implying that both hypothetical proteins may be expressed (see figure 6).

**Table 3:** Features of PCR-amplified mycobacterial rhomboids

	Primer	Species/Strain	Amplicon size (bp)	ORF (bp)	Amino acids	Accession number^h^	Protein ID
Orthologs of Rv0110 (rhomboid protease 1)	0110F 0110R	^a^H37Rv	967	855	284	HM453890	ADO17908
		
		^b^BCG	967	855	284	HM453894	ADO17912
		
		^c^JN55	967	855	284	HM453896	ADO17914
		
		^d^BN44	967	855	284	HM453892	ADO17910
	
	5036F 5036R	^e^SMR5	1000	891	296	HM453900	ADO17919

Orthologs of Rv1337 (rhomboid protease 2)	1337F 1337R	H37Rv	869	723	240	HM453891	ADO17909
		
		BCG	869	723	240	HM453895	ADO17913
		
		JN55	869	723	240	HM453897	ADO17915
		
		BN44	869	723	240	HM453897	ADO17911
	
	1554F 1554R	^f^SU-36800	954	672	223	HM453898	ADO17916
		
		^g^27	954	291 **(MAP2426c)**	72	HM453899	ADO17917
		
				444 **(MAP2425c)**	147	HM453899	ADO17917
	
	4904F 4904R	SMR5	845	738	245	HM453901	ADO17920

**^a^**: *M. tuberculosis*

**^b^**: *M. bovis*

**^c^**: *M. bovis *(cattle isolate)

**^d^**: *M. tuberculosis *(patient isolate)

**^e^**: *M. smegmatis *(streptomycin resistant derivative of MC^2^155)

**^f^**: *M. avium *(patient isolate)

**^g^**: *M. avium *subsp. *Paratuberculosis *(cattle isolate)

**^h^**: Accession numbers are for GenBank

Primer sequences are described in methods

### What are the lengths of MTC rhomboids?

In genome databases, the lengths for annotated sequences of rhomboids from genetically related mycobacteria vary, and initially we thought this reflected strain diversity. For instance, lengths for Rv0110 orthologs of MTC species are either 249 or 284 residues, while Rv1337 orthologs from the same species are 240 residues. In contrast, MT1378 (ortholog of Rv1337) of *M. tuberculosis *CDC 1551 is 227 amino acids, 13 residues shorter at the NH_2_-terminus. Thus, we aimed to validate the sizes of rhomboids from related strains/species. Genomic analyses at the rhomboid loci for the sequenced MTC genomes revealed that MTC rhomboid orthologs are 100% identical and are of equal length. Rhomboids were PCR-amplified from MTC with common primer sets for each ortholog (see methods), and sequencing data confirmed that MTC rhomboid orthologs are identical and are of the same size (284 residues for Rv0110 orthologs and 240 residues for Rv1337 orthologs). Rhomboid sequences were deposited in GenBank and accession numbers were assigned (see table 3).

### Putative gene clusters for mycobacterial rhomboids

To determine putative functional coupling between mycobacterial rhomboids and other genes, genes in clusters formed by mycobacterial rhomboids at the KEGG database [51] were analyzed. The gene cluster formed by Rv1337 was conserved across the genus and extended to other actinobacteria such as *Norcardia *and *Corynebacteria*. This cluster included 58 genes (Rv1311 to Rv1366, see additional file 5) of which some are essential and others are required for the growth of *M. tuberculosis *in macrophages [38], a necessary step during pathogenesis of the tubercle bacillus. Conversely, the Rv0110 orthologs formed clusters reflecting the genetic relatedness of mycobacteria. Thus, the orthologs from MTC species and *M. marinum *formed similar clusters consisting of 61 genes (Rv0080 to Rv0140, see additional file 6). These clusters also included essential genes and those required for survival of the tubercle bacillus in macrophage. However, MUL_4822 of genetically related *M. ulcerans *was not included in the MTC/*M. marinum *cluster, and formed a unique cluster consisting of only 19 genes (MUL_4791 to MUL_4824) with two genes upstream of the rhomboid (MUL_4823 and MUL_4824, see additional file 7). It is not certain whether this reflects functional divergence of MUL_4822 from Rv0110, in spite of evolutionary relatedness of *M. ulcerans *and MTC species.

The gene cluster of Rv0110 orthologs of *M. vanbaalenii*, *M. gilvum *and *Mycobacterium *species Jls, Kms and Mcs were also similar, and consisted of 48 genes (Mjls_5512 to Mjls_5559, see additional file 8), whose orthologs in MTC species are required for the growth of the tubercle bacillus in macrophages [38]. Conversely, the cluster for MAB_0026 of *M. abscessus *consisted of only three genes (MAB_0024, MAB_0025 and MAB_0026), shared with actinobacteria other than mycobacteria. Many MTC orthologs in the gene clusters of MUL_4822, Mjls_5529 and MAB_0026 are required for the growth of the bacillus in macrophages, the implication of which requires further study. There was no gene cluster formed by MSMEG_5036 of *M. smegmatis*. The essential genes in mycobacterial rhomboid gene clusters are described in additional file 9.

### Transcription analysis

Due to their ubiquity in eubacteria, we aimed to determine the expression of mycobacterial rhomboids in a preliminary fashion by screening for in vivo transcription. RT**- (**Reverse Transcriptase) PCRs amplified rhomboid cDNAs from mycobacterial mRNA, indicating that both copies of mycobacterial rhomboids are transcribed, and possibly expressed (see figure 6).

### Functional insights

#### Signal transduction and Metabolite transport

Since mycobacterial rhomboids contain rhomboid catalytic signatures, they may be functionally similar to *aarA *and *rho-1*, rescuing phenotypes associated with deletion of these genes in *P. stuartii *and *D. melanogaster *rhomboid mutants [52]. Due to their diverse functions, rhomboids appear good candidates for investigation in studies elucidating inter/intra-species/kingdom signaling mechanisms [29,53-55].

Furthermore, *gluP *(contains a rhomboid domain) of *B. subtilis *is involved in sugar transport [17,32], while aarA activates the TatA protein transporter in *P. stuartii *[31]. As such, the putative gene clusters for mycobacterial rhomboids contained putative metabolite transporters and transcriptional regulators. Since genes in clusters for transport and signal transduction genes tend to have similar roles [56], mycobacterial rhomboids may have such roles.

#### Roles in pathogenesis?

In a TraSH analysis by Rengarajan et al, Rv1337 was required for the survival of *M. tuberculosis *H37Rv in macrophages [38], a necessary step during the development of TB. The genome wide conservation of Rv1337 alludes to a possibly important protein. The pathogenesis of *M. ulcerans*, (the only mycobacterium lacking the Rv1337 ortholog) is known and it culminates in skin ulcerations caused by the plasmid encoded polyketide toxin -mycolactone [4,40,44,57]. Buruli ulcer contrasts with the tuberculous nature of lesions formed by many pathogenic mycobacteria, whose pathogenesis is not well understood and remains a vast field of study.

#### Moonlighting properties?

It is possible to predict functional coupling between genes based on conservation of gene clusters among genomes [56,58]. Since proteins encoded by conserved gene pairs appear to interact physically [58], the evolutionary conservation of the Rv1337 genome arrangement might have functional implications. mur1 is a moonlighting protein (ability to perform multiple independent functions [59]) that exhibits both racemization and DNA gyrase activities [59]. Since rhomboids are known for diverse functions, the proximity of Rv1337 orthologs with a moonlighting protein makes them suspects for moonlighting properties.

## Conclusions

### Mycobacterial rhomboids have different evolutionary history

The two mycobacterial rhomboids are phylogenetically distinct and could have been acquired independently. The mycobacterial orthologs of Rv0110 (rhomboid protease 1) appear to be under evolutionary pressure; hence they were lost in the MAC species and *M. leprae*. These orthologs represent prokaryotic rhomboids whose progenitor may be the ancestor for eukaryotic rhomboids. The Rv1337 (rhomboid protease 2) mycobacterial orthologs appear more stable and are conserved nearly in all mycobacteria, possibly alluding to their importance in mycobacteria.

MAP2425c and MAP2426c provide the first evidence of a split rhomboid contrasting whole orthologs of genetically related species.

### Mycobacterial rhomboids are active rhomboid proteases

Mycobacterial rhomboids are active rhomboid proteases, with the active site being stabilized by Phe. Although valuable insights to the roles of rhomboids are provided, the data herein only lays a foundation for future investigations for the roles of rhomboids in mycobacteria.

## Methods

### Strains and cultures

*Mycobacterium smegmatis *SMR5 (streptomycin resistant derivative of MC^2^155) and *M. avium *(patient isolate SU-36800) were obtained from the Joint Clinical Research Center (JCRC), Kampala, Uganda. The streptomycin resistant derivatives of *M. tuberculosis *H37Rv and *M. bovis *BCG were provided by Dr. Peter Sander, University of Zurich, Switzerland. *M. tuberculosis *BN44 and *M. bovis *JN55 are characterized clinical isolates [60,61]. *M. avium *subsp. *Paratuberculosis *was provided by Dr. Julius B. Okuni, Faculty of Veterinary Medicine, Makerere University. *M. smegmatis *was cultured in LB/0.05% Tween 80 containing 200 μg/ml streptomycin. MTC and MAC strains were cultured in middlebrook 7H9 or 7H10 (supplemented with mycobactin for MAP cultures).

### PCR conditions

Chromosomal DNA was extracted from mycobacteria by boiling heat-killed cells for 10 min and centrifuging briefly at 5000 g to obtain the supernatant containing DNA. Amplification reactions contained 20 pmol each of the rhomboid specific forward and reverse primers (see below), 1.5 U of high fidelity *Taq *polymerase (Roche Applied Science, Mannheim, Germany), Custom PCR Master Mix (Thermo Scientific, Surry, UK), ~200 ng template DNA and nuclease-free water in a reaction volume of 10 μL. The reactions were performed in a Peltier thermocycler (MJ Research, Waterman, MA, USA) at the following conditions: initial denaturation at 94°C for 5 min, followed by 30 cycles each consisting of 94°C, 0.5 min; 60°C, 0.3 min & 72°C, 1 min, with a final extension at 72°C for 10 min. Following amplification, the amplicons were purified with QIAquick PCR purification kit (Qiagen, Hilden, Germany) and sequenced at ACGT (Wheeling, IL, USA). After analyzing with BioEdit software and BLAST algorithm for similarity searches, rhomboid sequences were deposited in the GenBank database (see table 3 for accession numbers).

The following primers were used: 0110F, 5'-ATATTCGGCTTCGCCGGAACC-3' (forward) and 0110R, 5'-ACGCGAAGACAAGCGGCTATC-3' (reverse) for MTC Rv0110 orthologs; 1337F, 5' ACGCCGGGTGGAAGTATCTG-3' (forward) and 1337R, 5'-CCGACGCCGGAATCAAAGACTC-3' (reverse) for MTC Rv1337 orthologs. For MAC species, primer pair 1554F, 5'-TCGACGGTGACACCGTGTTC-3' (forward) and 1554R, 5'-TGCCGAGCTCATGTCTTGGG-3' (reverse) was used. For *M. smegmatis*, primer pairs 5036F, 5'-ACGGCCGGGTGAGACAAATC-3' (forward) and 5036R, 5'-TGGACCCGGACAACATCCTG-3' (reverse) for homolog MSMEG_5036; 4904F, 5'-ACGCCGGATGGAAGTATCTG-3' (forward) and 4904R, 5'-ACACCGGAATCGAAGATCCC-3' (reverse) for homolog MSMEG_4904 were used. Primers were synthesized by IDT (Leuven, Belgium).

### Transcription assays

mRNA was purified from mycobacteria with the Oligotex mRNA mini kit (Qiagen, Hilden, Germany) and ~60 ng/μl (in 15 μl) mRNA used as template for cDNA synthesis. Reverse Transcriptase-PCRs were performed with the Titan One Tube RT-PCR System (Roche Applied Science, Mannheim, Germany) to amplify Rv0110 and Rv1337 cDNAs in separate reactions. Except for the initial cDNA synthesis step (50°C for 30 min), PCR conditions were similar to those described above. RT-PCRs were repeated with primers (1337int1: TGGACGTCAACGGCATCAG, forward, and 1337int2: CCAGCCCAATGACGATATCCC, reverse) that amplify an internal fragment (~350 bp) of Rv1337 orthologs.

### Bioinformatic analyses

#### Identification of rhomboids in mycobacteria

Rhomboid sequences for rho-7 [GenBank: NP_523704.1] of *D. melanogaster*, PARL [GenBank: NP_061092.3] of human, glpG [GenBank: AAA23890] of *E. coli *and aarA [GenBank: L28755] of *P. stuartii *were obtained from GenBank [62]. These sequences were used as queries in BLAST-searches for rhomboid homologs from an array of mycobacterial genome databases: "tuberculist" [63], GIB-DDBJ [64] and J. Craig Venter institute [65].

#### Sequence analysis

The similarity between mycobacterial rhomboids was determined using specialized BLAST bl2seq for comparing two or more sequences [66]. Multiple sequence alignments were performed with ClustalW [67] or MUSCLE [68]. Mycobacterial rhomboids were examined for the presence of rhomboid family domains and catalytic signatures (GxSx). The TMH predictions were done using the TMHMM Server v. 2.0 [69]. The data generated was fed into the TMRPres2D [70] database to generate high resolution images. Cellular localization signals were predicted using TargetP 1.1 server [71].

#### Phylogenetic analysis

Phylogenetic analysis was conducted using MEGA4 software [72]. The evolutionary history of mycobacterial rhomboids was determined using the Neighbor-Joining method. The percentage of replicate trees in which the associated taxa clustered together was determined using the Bootstrap test (1000 replicates). The evolutionary distances were computed using the Poisson correction method and are in the units of the number of amino acid substitutions per site. All positions containing gaps and missing data were eliminated from the dataset (complete deletion option). For comparison of evolutionary history, trees were also constructed using "Minimum Evolution" and "Maximum Parsimony".

#### Functional predictions

To predict possible roles for mycobacterial rhomboids, sequences were analyzed at the KEGG database [51] for the genome arrangement, presence of extra protein domains, nature of gene clusters, orthologs and paralogs. Other parameters used to glean functions from mycobacterial rhomboid sequences included analyzing their topologies. To predict functional relatedness among genes within mycobacterial rhomboid clusters, sequences in the clusters were aligned by ClustalW, and Neighbor-Joining trees deduced using default settings.

## Abbreviations

BLAST: Basic Local Alignment Search Tool; GIB-DDBJ: Genome information Broker-DNA Data Bank of Japan; ESAT-6: Early Secreted Antigenic Target 6 kDa protein; iRhoms: inactive rhomboids; KEGG: Kyoto Encyclopedia of Genes and Genomes; LB: Luria Bertani; MAC: *Mycobacterium avium *Complex; MAP: *Mycobacterium avium *subspecies *Paratuberculosis*; MTC: *Mycobacterium tuberculosis *Complex; MUSCLE: Multiple Sequence Comparison by Log-Expectation; NTM: None-tuberculous mycobacteria; ORF: Open Reading Frame; PARL: Presenilin-associated rhomboid-like; PDIM: Phthiocerol Dimycocerosate; RT-PCR: Reverse Transcriptase Polymerase Chain Reaction; SNP: Single Nucleotide Polymorphism; TraSH: Transposon Site Hybridization; TMH: Transmembrane helice;

## Competing interests

The authors declare that they have no competing interests.

## Authors' contributions

DPK and MLJ conceived and designed the study, supervised by MLJ. DPK performed the bioinformatics and wrote the manuscript in partial fulfillment for his PhD. MO purified mRNA and performed the RT-PCRs. The other authors read and critiqued the manuscript. All authors read and approved the final manuscript.

## Supplementary Material

Additional file 1 **The topology and location of catalytic residues in mycobacterial rhomboid protease 1 (Rv0110 orthologs)**. As in rho-1, the catalytic residues are located in TMH4 (Gly199 and Ser201) and TMH6 (His254), while His145, His150 and Asn154 are in TMH2. Click here for file

Additional file 2 **The topology and location of catalytic residues in rho-1 of Drosophila**. As in mycobacterial rhomboid protease 1, the catalytic residues are located in TMH4 (Gly199 and Ser201) and TMH6 (His254), while His145, His150 and Asn154 are in TMH2. Click here for file

Additional file 3 **The topology and location of catalytic residues in mycobacterial rhomboid protease 2 (Rv1337 orthologs)**. The orthologs of pathogenic mycobcateria formed six TMHs, with catalytic residues in TMH4 (Gly199 and Ser201) and TMH6 (His254). His145, His150 and Asn154 are located in TMH2 as in rhomboid protease-1 (Rv0110 orthologs). Click here for file

Additional file 4 **The topology and location of catalytic residues in mycobacterial rhomboid protease 2 (Rv1337 orthologs) of nonpathogenic mycobacteria**. These rhomboids formed five TMHs, with catalytic residues in TMH3 (Gly199 and Ser201) and TMH5 (His254), while His145, His150 and Asn154 are outside the TMHs (boxed). Click here for file

Additional file 5 **ClustalW-Neighbor Joining analysis of the genes in Rv1337 cluster**. Boxed (blue) are the genes that grouped with Rv1337. Essential genes in this clade are Rv1327c, Rv1327c, Rv1331, Rv1340 and Rv1344. Click here for file

Additional file 6 **ClustalW-Neighbor Joining analysis of the genes in Rv0110 cluster**. Boxed (blue) are the essential genes in that grouped with Rv0110 (Rv0118c, Rv0127, Rv0107c, Rv0116c, Rv0121c, Rv0132c, Rv0133 and Rv0139). Click here for file

Additional file 7 **ClustalW-Neighbor Joining analysis of the genes in MUL4822 cluster**. Boxed (blue) are the genes that grouped with MUL4822. Several of the MTC orthologs in this clade are essential for the growth of *M. tuberculosis *in macrophages. Click here for file

Additional file 8 **ClustalW-Neighbor Joining analysis of the genes in Mjls5529 cluster**. Boxed (blue) are the genes that grouped with Mjls5529, whose homologs are essential in *M. tuberculosis*. Several of the MTC orthologs in this clade are essential for the growth of *M. tuberculosis *in macrophages. Click here for file

Additional file 9 **The essential genes in mycobacterial rhomboid gene clusters (doc)**. **^a^: **According to Sassetti et al [37] and Rengarajan et al [38]. **^1^: **Essential (for optimal growth). **^2^: **Required for growth in macrophage. **^3^: **Mutation slows growth. Click here for file

## References

[B1] Euzéby JP List of Prokaryotic names with Standing in Nomenclature http://www.bacterio.cict.fr/m/mycobacterium.html

[B2] Cole ST Brosch R Parkhill J Garnier T Churcher C Harris D Gordon SV Eiglmeier K Gas S Barry CE Deciphering the biology of Mycobacterium tuberculosis from the complete genome sequence Nature 1998 393 6685 537 544 10.1038/31159 9634230

[B3] Cole ST Eiglmeier K Parkhill J James KD Thomson NR Wheeler PR Honore N Garnier T Churcher C Harris D Massive gene decay in the leprosy bacillus Nature 2001 409 6823 1007 1011 10.1038/35059006 11234002

[B4] Demangel C Stinear TP Cole ST Buruli ulcer: reductive evolution enhances pathogenicity of Mycobacterium ulcerans Nat Rev Microbiol 2009 7 1 50 60 10.1038/nrmicro2077 19079352

[B5] Bannantine JP Barletta RG Stabel JR Paustian ML Kapur V Application of the Genome Sequence to Address Concerns That Mycobacterium avium Subspecies Paratuberculosis Might Be a Foodborne Pathogen Foodborne Pathogens and Disease 2004 1 1 3 15 10.1089/153531404772914419 15992257

[B6] Rubin DS Rahal JJ Mycobacterium-avium complex Infect Dis Clin North Am 1994 8 2 413 426 8089468

[B7] Valentin-Weigand P Goethe R Pathogenesis of Mycobacterium avium subspecies paratuberculosis infections in ruminants: still more questions than answers Microbes Infect 1999 1 13 1121 1127 10.1016/s1286-4579(99)00203-8 10572316

[B8] Ventura M Canchaya C Tauch A Chandra G Fitzgerald GF Chater KF van Sinderen D Genomics of Actinobacteria: tracing the evolutionary history of an ancient phylum Microbiol Mol Biol Rev 2007 71 3 495 548 10.1128/MMBR.00005-07 PMC2168647 17804669

[B9] Khan AA Kim SJ Paine DD Cerniglia CE Classification of a polycyclic aromatic hydrocarbon-metabolizing bacterium, Mycobacterium sp. strain PYR-1, as Mycobacterium vanbaalenii sp. nov Int J Syst Evol Microbiol 2002 52 Pt 6 1997 2002 10.1099/00207713-52-6-1997 12508859

[B10] Brodin P Rosenkrands I Andersen P Cole ST Brosch R ESAT-6 proteins: protective antigens and virulence factors? Trends in microbiology 2004 12 11 500 508 10.1016/j.tim.2004.09.007 15488391

[B11] Chen JM Islam ST Ren H Liu J Differential productions of lipid virulence factors among BCG vaccine strains and implications on BCG safety Vaccine 2007 25 48 8114 8122 10.1016/j.vaccine.2007.09.041 17954004

[B12] Smith I Mycobacterium tuberculosis pathogenesis and molecular determinants of virulence Clin Microbiol Rev 2003 16 3 463 496 10.1128/CMR.16.3.463-496.2003 PMC164219 12857778

[B13] McDevitt D Rosenberg M Exploiting genomics to discover new antibiotics Trends Microbiol 2001 9 12 611 617 10.1016/s0966-842x(01)02235-1 11728875

[B14] Traag BA Driks A Stragier P Bitter W Broussard G Hatfull G Chu F Adams KN Ramakrishnan L Losick R Do mycobacteria produce endospores? Proc Natl Acad Sci USA 107 2 878 881 10.1073/pnas.0911299107 PMC2818926 20080769

[B15] Bansal AK Bioinformatics in microbial biotechnology--a mini review Microb Cell Fact 2005 4 1 19 10.1186/1475-2859-4-19 PMC1182391 15985162

[B16] Godreuil S Tazi IL Bañuls AL Pulmonary Tuberculosis and Mycobacterium Tuberculosis: Modern Molecular Epidemiology and Perspectives http://media.wiley.com/product_data/excerpt/28/04716573/0471657328.pdf

[B17] Freeman M Rhomboid proteases and their biological functions Annu Rev Genet 2008 42 191 210 10.1146/annurev.genet.42.110807.091628 18605900

[B18] Wasserman JD Urban S Freeman M A family of rhomboid-like genes: Drosophila rhomboid-1 and roughoid/rhomboid-3 cooperate to activate EGF receptor signaling Genes Dev 2000 14 13 1651 1663 PMC316740 10887159

[B19] Koonin EV Makarova KS Rogozin IB Davidovic L Letellier MC Pellegrini L The rhomboids: a nearly ubiquitous family of intramembrane serine proteases that probably evolved by multiple ancient horizontal gene transfers Genome Biol 2003 4 3 R19 10.1186/gb-2003-4-3-r19 PMC153459 12620104

[B20] Urban S Rhomboid proteins: conserved membrane proteases with divergent biological functions Genes Dev 2006 20 22 3054 3068 10.1101/gad.1488606 17114579

[B21] Baker RP Wijetilaka R Urban S Two Plasmodium rhomboid proteases preferentially cleave different adhesins implicated in all invasive stages of malaria PLoS Pathog 2006 2 10 e113 10.1371/journal.ppat.0020113 PMC1599764 17040128

[B22] Carruthers VB Proteolysis and Toxoplasma invasion Int J Parasitol 2006 36 5 595 600 10.1016/j.ijpara.2006.02.008 16600244

[B23] Dowse TJ Pascall JC Brown KD Soldati D Apicomplexan rhomboids have a potential role in microneme protein cleavage during host cell invasion Int J Parasitol 2005 35 7 747 756 10.1016/j.ijpara.2005.04.001 15913633

[B24] Srinivasan P Coppens I Jacobs-Lorena M Distinct roles of Plasmodium rhomboid 1 in parasite development and malaria pathogenesis PLoS Pathog 2009 5 1 e1000262 10.1371/journal.ppat.1000262 PMC2607553 19148267

[B25] Brossier F Jewett TJ Sibley LD Urban S A spatially localized rhomboid protease cleaves cell surface adhesins essential for invasion by Toxoplasma Proc Natl Acad Sci USA 2005 102 11 4146 4151 10.1073/pnas.0407918102 PMC554800 15753289

[B26] Yan Z Zou H Tian F Grandis JR Mixson AJ Lu PY Li LY Human rhomboid family-1 gene silencing causes apoptosis or autophagy to epithelial cancer cells and inhibits xenograft tumor growth Mol Cancer Ther 2008 7 6 1355 1364 10.1158/1535-7163.MCT-08-0104 PMC3426753 18524845

[B27] Zou H Thomas SM Yan ZW Grandis JR Vogt A Li LY Human rhomboid family-1 gene RHBDF1 participates in GPCR-mediated transactivation of EGFR growth signals in head and neck squamous cancer cells FASEB J 2009 23 2 425 432 10.1096/fj.08-112771 PMC2638965 18832597

[B28] Waters CM Bassler BL Quorum sensing: cell-to-cell communication in bacteria Annu Rev Cell Dev Biol 2005 21 319 346 10.1146/annurev.cellbio.21.012704.131001 16212498

[B29] Federle MJ Bassler BL Interspecies communication in bacteria J Clin Invest 2003 112 9 1291 1299 10.1172/JCI20195 PMC228483 14597753

[B30] Rather PN Orosz E Characterization of aarA, a pleiotrophic negative regulator of the 2'-N-acetyltransferase in Providencia stuartii J Bacteriol 1994 176 16 5140 5144 10.1128/jb.176.16.5140-5144.1994 PMC196357 8051030

[B31] Mesak LR Mesak FM Dahl MK Expression of a novel gene, gluP, is essential for normal Bacillus subtilis cell division and contributes to glucose export BMC Microbiol 2004 4 13 10.1186/1471-2180-4-13 PMC408461 15050034

[B32] Clemmer KM Sturgill GM Veenstra A Rather PN Functional characterization of Escherichia coli GlpG and additional rhomboid proteins using an aarA mutant of Providencia stuartii J Bacteriol 2006 188 9 3415 3419 10.1128/JB.188.9.3415-3419.2006 PMC1447467 16621838

[B33] Wu Z Yan N Feng L Oberstein A Yan H Baker RP Gu L Jeffrey PD Urban S Shi Y Structural analysis of a rhomboid family intramembrane protease reveals a gating mechanism for substrate entry Nat Struct Mol Biol 2006 13 12 1084 1091 10.1038/nsmb1179 17099694

[B34] Lieberman RL Wolfe MS Membrane-embedded protease poses for photoshoot Proc Natl Acad Sci USA 2007 104 2 401 402 10.1073/pnas.0610236103 PMC1766396 17213330

[B35] Lemberg MK Freeman M Functional and evolutionary implications of enhanced genomic analysis of rhomboid intramembrane proteases Genome Res 2007 17 11 1634 1646 10.1101/gr.6425307 PMC2045146 17938163

[B36] Sassetti CM Boyd DH Rubin EJ Comprehensive identification of conditionally essential genes in mycobacteria Proc Natl Acad Sci USA 2001 98 22 12712 12717 10.1073/pnas.231275498 PMC60119 11606763

[B37] Sassetti CM Boyd DH Rubin EJ Genes required for mycobacterial growth defined by high density mutagenesis Mol Microbiol 2003 48 1 77 84 10.1046/j.1365-2958.2003.03425.x 12657046

[B38] Rengarajan J Bloom BR Rubin EJ Genome-wide requirements for Mycobacterium tuberculosis adaptation and survival in macrophages Proc Natl Acad Sci USA 2005 102 23 8327 8332 10.1073/pnas.0503272102 PMC1142121 15928073

[B39] McEvoy CR van Helden PD Warren RM Gey NC van Pittius Evidence for a rapid rate of molecular evolution at the hypervariable and immunogenic Mycobacterium tuberculosis PPE38 gene region BMC Evol Biol 2009 9 237 10.1186/1471-2148-9-237 PMC2758852 19769792

[B40] Yip MJ Porter JL Fyfe JA Lavender CJ Portaels F Rhodes M Kator H Colorni A Jenkin GA Stinear T Evolution of Mycobacterium ulcerans and other mycolactone-producing mycobacteria from a common Mycobacterium marinum progenitor J Bacteriol 2007 189 5 2021 2029 10.1128/JB.01442-06 PMC1855710 17172337

[B41] Balakirev ES Ayala FJ Pseudogenes: are they "junk" or functional DNA? Annu Rev Genet 2003 37 123 151 10.1146/annurev.genet.37.040103.103949 14616058

[B42] Piehler AP Hellum M Wenzel JJ Kaminski E Haug KB Kierulf P Kaminski WE The human ABC transporter pseudogene family: Evidence for transcription and gene-pseudogene interference BMC Genomics 2008 9 165 10.1186/1471-2164-9-165 PMC2329642 18405356

[B43] Piehler AP Wenzel JJ Olstad OK Haug KB Kierulf P Kaminski WE The human ortholog of the rodent testis-specific ABC transporter Abca17 is a ubiquitously expressed pseudogene (ABCA17P) and shares a common 5' end with ABCA3 BMC Mol Biol 2006 7 28 10.1186/1471-2199-7-28 PMC1579226 16968533

[B44] Stinear TP Seemann T Pidot S Frigui W Reysset G Garnier T Meurice G Simon D Bouchier C Ma L Reductive evolution and niche adaptation inferred from the genome of Mycobacterium ulcerans, the causative agent of Buruli ulcer Genome Res 2007 17 2 192 200 10.1101/gr.5942807 PMC1781351 17210928

[B45] Stinear TP Seemann T Harrison PF Jenkin GA Davies JK Johnson PD Abdellah Z Arrowsmith C Chillingworth T Churcher C Insights from the complete genome sequence of Mycobacterium marinum on the evolution of Mycobacterium tuberculosis Genome Res 2008 18 5 729 741 10.1101/gr.075069.107 PMC2336800 18403782

[B46] Stinear TP Jenkin GA Johnson PD Davies JK Comparative genetic analysis of Mycobacterium ulcerans and Mycobacterium marinum reveals evidence of recent divergence J Bacteriol 2000 182 22 6322 6330 10.1128/jb.182.22.6322-6330.2000 PMC94777 11053375

[B47] Koonin EV Orthologs, paralogs, and evolutionary genomics Annu Rev Genet 2005 39 309 338 10.1146/annurev.genet.39.073003.114725 16285863

[B48] Joshi T Xu D Quantitative assessment of relationship between sequence similarity and function similarity BMC Genomics 2007 8 222 10.1186/1471-2164-8-222 PMC1949826 17620139

[B49] Dowse TJ Soldati D Rhomboid-like proteins in Apicomplexa: phylogeny and nomenclature Trends Parasitol 2005 21 6 254 258 10.1016/j.pt.2005.04.009 15922242

[B50] Gaur RK Natekar GA Prokaryotic and eukaryotic integral membrane proteins have similar architecture Mol Biol Rep 37 3 1247 1251 10.1007/s11033-009-9497-3 19267253

[B51] KEGG Kyoto Encyclopedia of Genes and Genomes http://www.genome.jp/kegg/

[B52] Gallio M Sturgill G Rather P Kylsten P A conserved mechanism for extracellular signaling in eukaryotes and prokaryotes Proc Natl Acad Sci USA 2002 99 19 12208 12213 10.1073/pnas.192138799 PMC129423 12221285

[B53] Hughes DT Sperandio V Inter-kingdom signalling: communication between bacteria and their hosts Nat Rev Microbiol 2008 6 2 111 120 10.1038/nrmicro1836 PMC2667375 18197168

[B54] Lowery CA Dickerson TJ Janda KD Interspecies and interkingdom communication mediated by bacterial quorum sensing Chem Soc Rev 2008 37 7 1337 1346 10.1039/b702781h 18568160

[B55] Ryan RP Dow JM Diffusible signals and interspecies communication in bacteria Microbiology 2008 154 Pt 7 1845 1858 10.1099/mic.0.2008/017871-0 18599814

[B56] Overbeek R Fonstein M D'Souza M Pusch GD Maltsev N The use of gene clusters to infer functional coupling Proc Natl Acad Sci USA 1999 96 6 2896 2901 10.1073/pnas.96.6.2896 PMC15866 10077608

[B57] Portaels F Meyers WM Ablordey A Castro AG Chemlal K de Rijk P Elsen P Fissette K Fraga AG Lee R First cultivation and characterization of Mycobacterium ulcerans from the environment PLoS Negl Trop Dis 2008 2 3 e178 10.1371/journal.pntd.0000178 PMC2268003 18365032

[B58] Narayan A Sachdeva P Sharma K Saini AK Tyagi AK Singh Y Serine threonine protein kinases of mycobacterial genus: phylogeny to function Physiol Genomics 2007 29 1 66 75 10.1152/physiolgenomics.00221.2006 17148687

[B59] Sengupta S Ghosh S Nagaraja V Moonlighting function of glutamate racemase from Mycobacterium tuberculosis: racemization and DNA gyrase inhibition are two independent activities of the enzyme Microbiology 2008 154 Pt 9 2796 2803 10.1099/mic.0.2008/020933-0 18757813

[B60] Asiimwe BB Asiimwe J Kallenius G Ashaba FK Ghebremichael S Joloba M Koivula T Molecular characterisation of Mycobacterium bovis isolates from cattle carcases at a city slaughterhouse in Uganda Vet Rec 2009 164 21 655 658 10.1136/vr.164.21.655 19465755

[B61] Asiimwe BB Koivula T llenius G Huard RC Ghebremichael S Asiimwe J Joloba ML Mycobacterium tuberculosis Uganda genotype is the predominant cause of TB in Kampala, Uganda The International Journal of Tuberculosis and Lung Disease 2008 12 386 391 18371263

[B62] NCBI National Center for Biotechnological Information http://www.ncbi.nlm.nih.gov/

[B63] TubercuList-GenoList http://genolist.pasteur.fr/TubercuList/

[B64] GIB Genome Information Broker http://gib.genes.nig.ac.jp/

[B65] JCVI J Craig Venter Institute http://cmr.jcvi.org/cgi-bin/CMR/CmrHomePage.cgi

[B66] Specialized BLAST: Align two or more sequences http://blast.ncbi.nlm.nih.gov/Blast.cgi

[B67] ClustalW http://www.ebi.ac.uk/clustalw/

[B68] MUSCLE MUltiple Sequence Comparison by Log-Expectation http://www.ebi.ac.uk/Tools/muscle/index.html

[B69] ExPASy Proteomics Server http://www.cbs.dtu.dk/services/TMHMM-2.0/

[B70] TMRPres2D Tool http://bioinformatics.biol.uoa.gr/TMRPres2D

[B71] ExPASY Tools http://www.cbs.dtu.dk/services/TargetP/

[B72] MEGA 4 Molecular Evolutionary Genetics Analysis http://www.megasoftware.net/

