# Supplementary figures and images for: Rhomboid homologs in mycobacteria: insights from phylogeny and genomic analysis
Source: BMC Microbiol. 2010 Oct 29;10:272. doi: 10.1186/1471-2180-10-272 (PMC2989971; doi:10.1186/1471-2180-10-272)

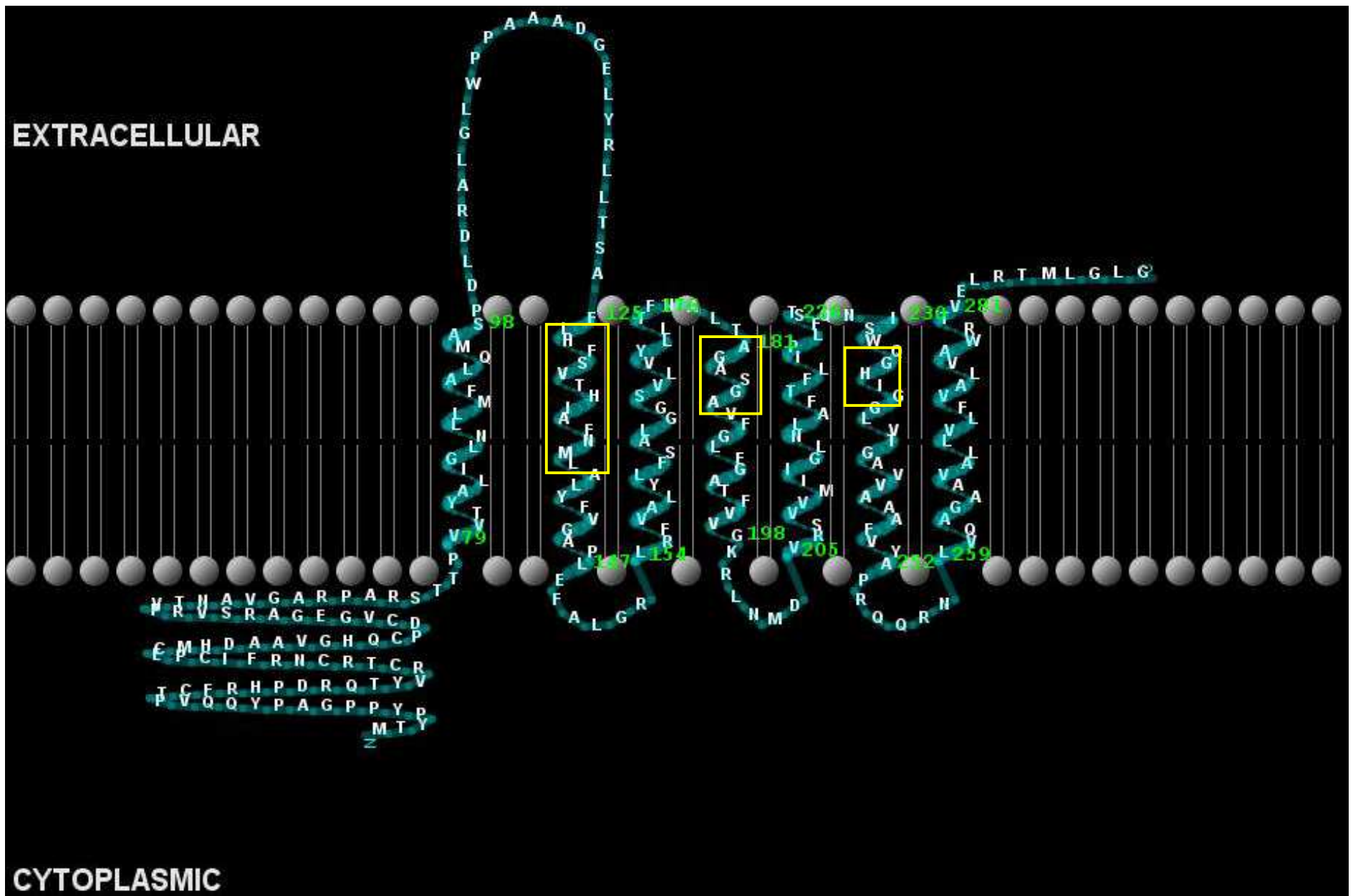

Supplement: Additional file 1 — The topology and location of catalytic residues in mycobacterial rhomboid protease 1 (Rv0110 orthologs). As in rho-1, the catalytic residues are located in TMH4 (Gly199 and Ser201) and TMH6 (His254), while His145, His150 and Asn154 are in TMH2. [file 1471-2180-10-272-S1.PDF]

EXTRACELLULAR

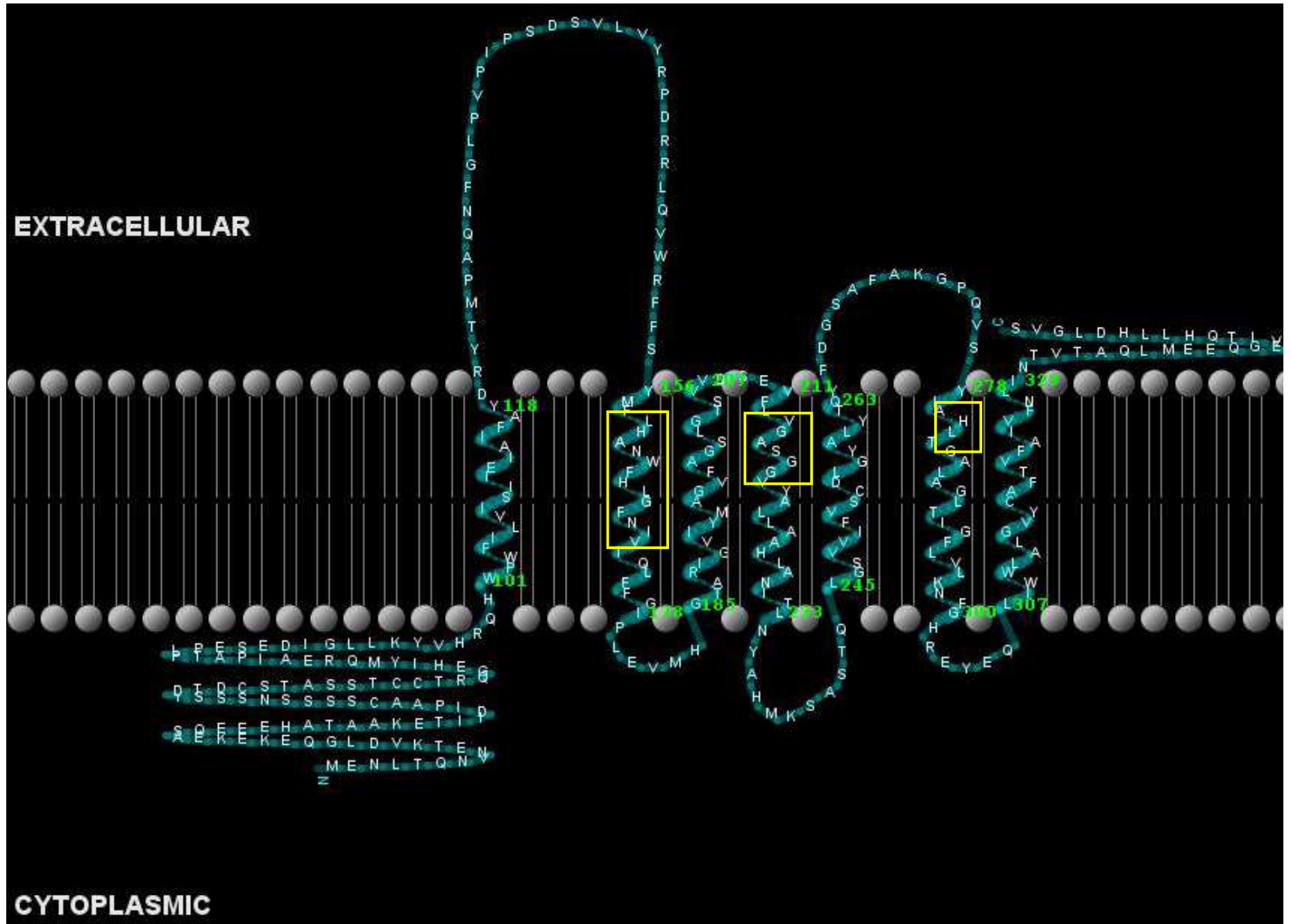

Supplement: Additional file 2 — The topology and location of catalytic residues in rho-1 of Drosophila. As in mycobacterial rhomboid protease 1, the catalytic residues are located in TMH4 (Gly199 and Ser201) and TMH6 (His254), while His145, His150 and Asn154 are in TMH2. [file 1471-2180-10-272-S2.PDF]

**EXTRACELLULAR**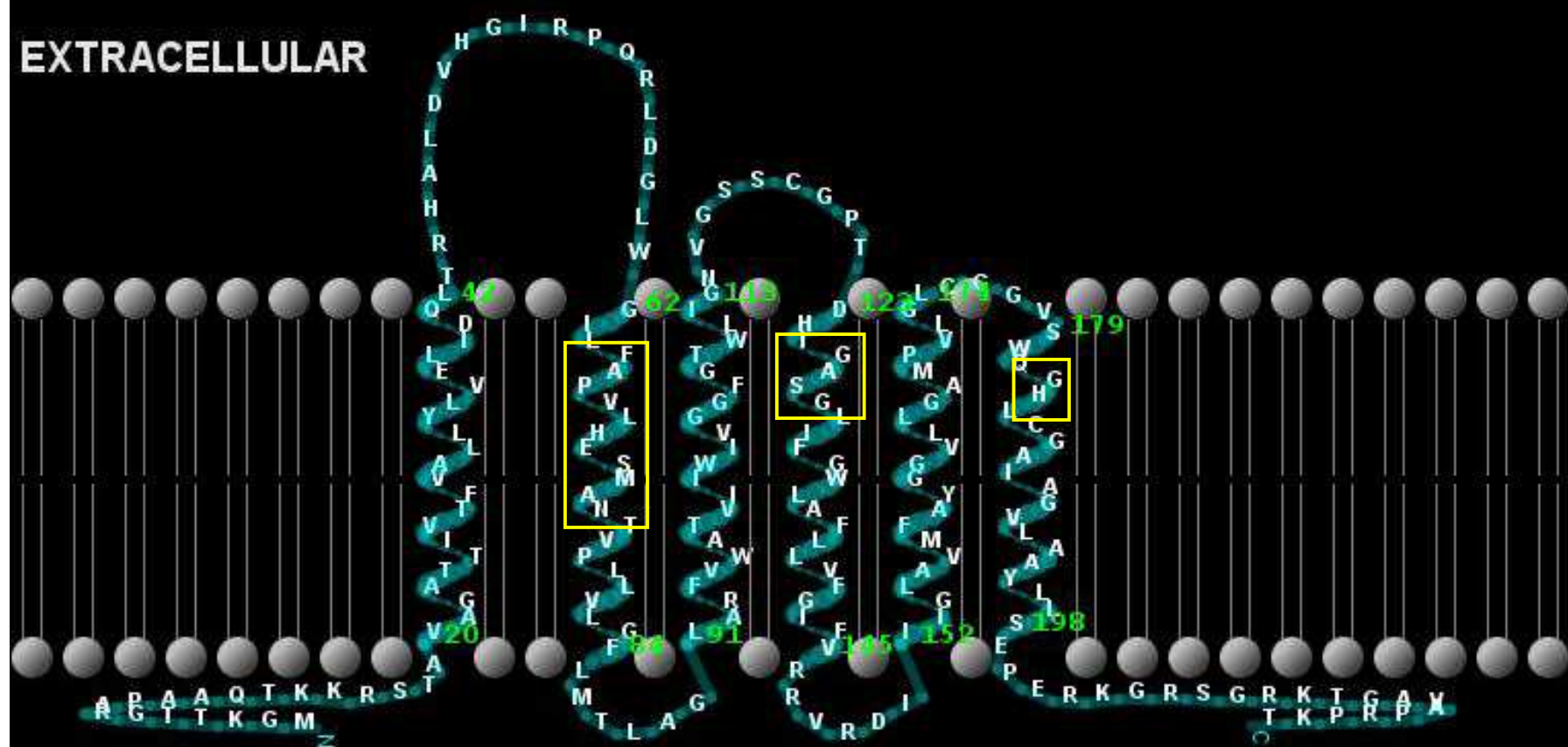

**CYTOPLASMIC**

Supplement: Additional file 3 — The topology and location of catalytic residues in mycobacterial rhomboid protease 2 (Rv1337 orthologs). The orthologs of pathogenic mycobcateria formed six TMHs, with catalytic residues in TMH4 (Gly199 and Ser201) and TMH6 (His254). His145, His150 and Asn154 are located in TMH2 as in rhomboid protease-1 (Rv0110 orthologs). [file 1471-2180-10-272-S3.PDF]

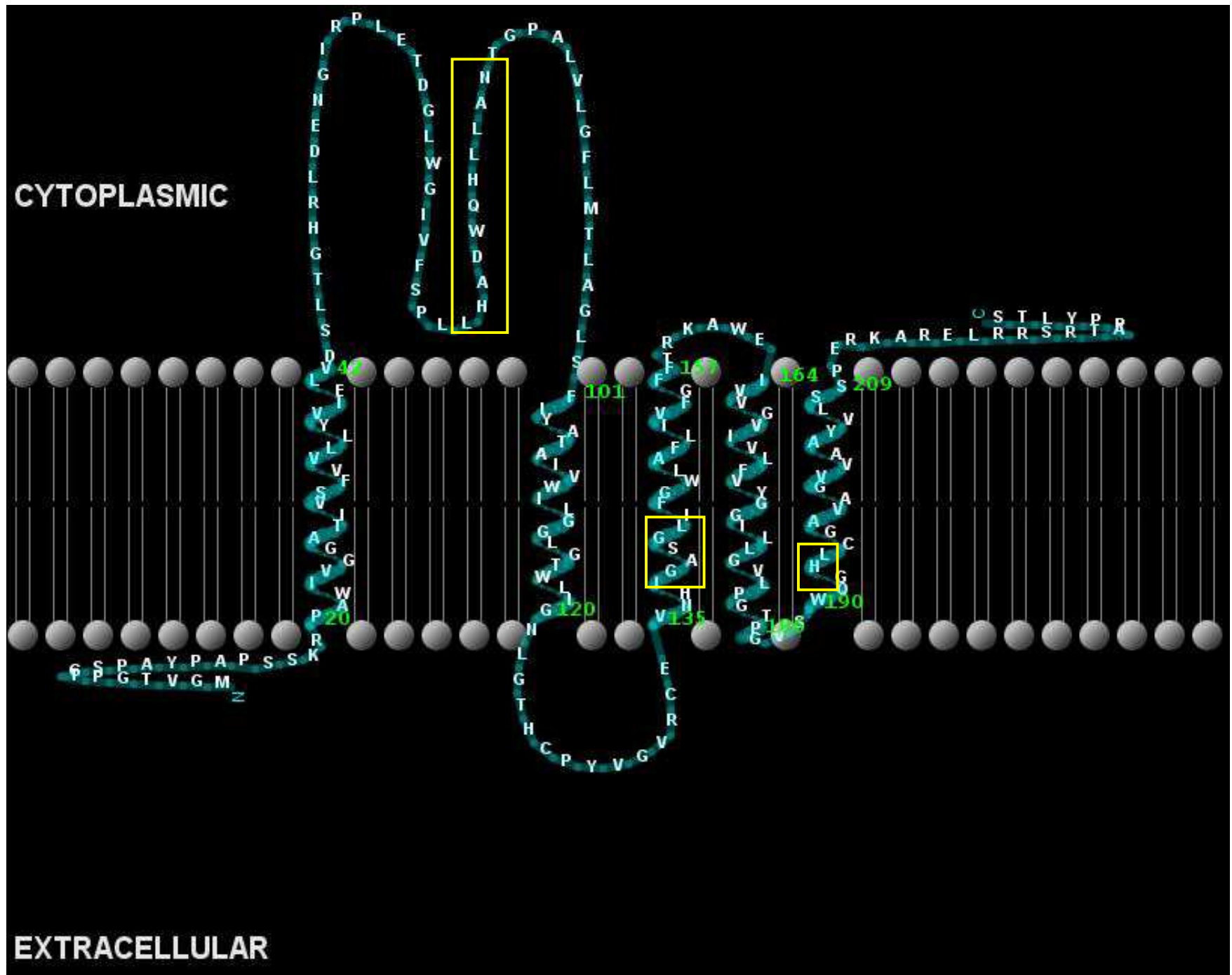

Supplement: Additional file 4 — The topology and location of catalytic residues in mycobacterial rhomboid protease 2 (Rv1337 orthologs) of nonpathogenic mycobacteria. These rhomboids formed five TMHs, with catalytic residues in TMH3 (Gly199 and Ser201) and TMH5 (His254), while His145, His150 and Asn154 are outside the TMHs (boxed). [file 1471-2180-10-272-S4.PDF]

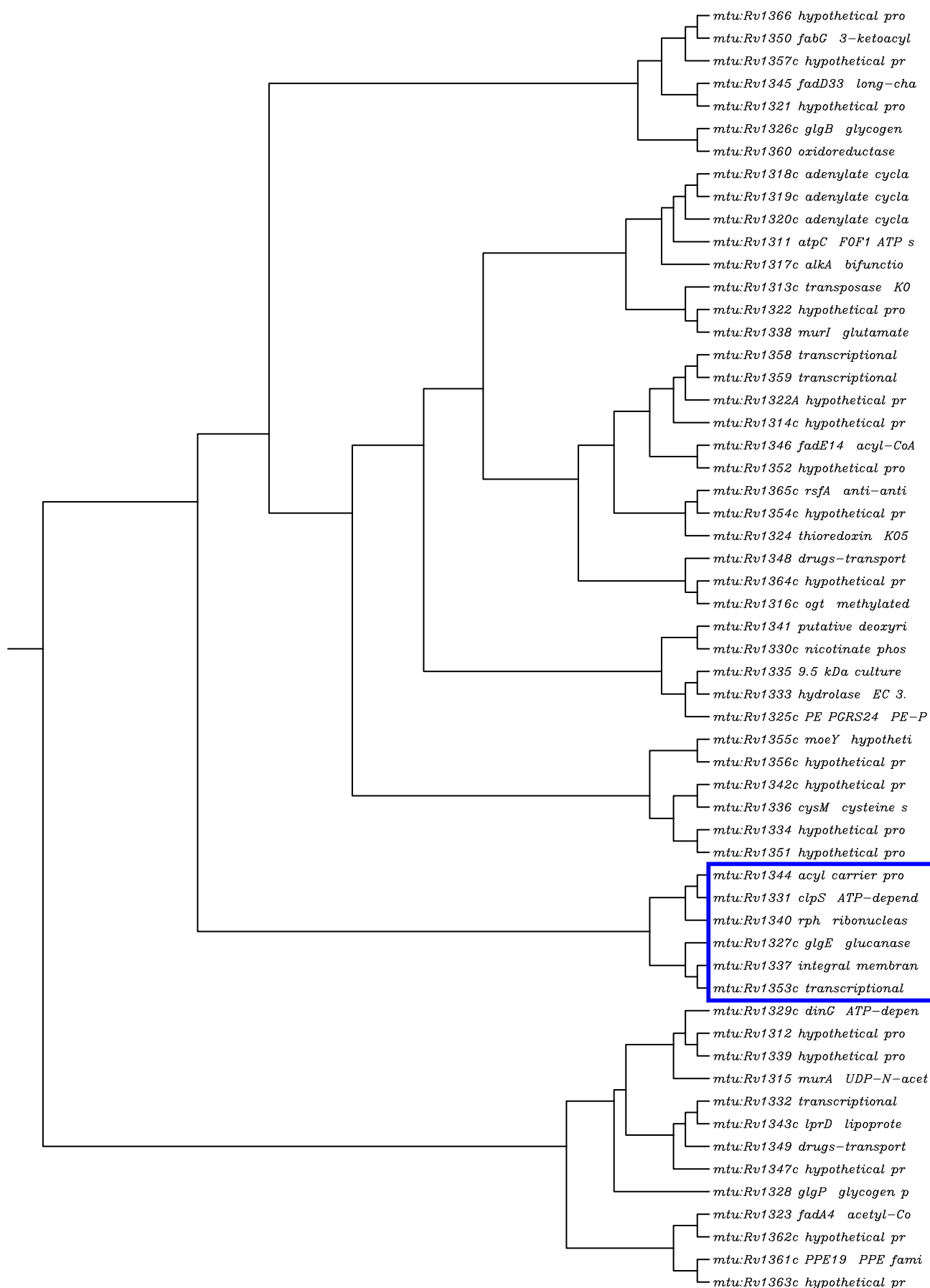

Supplement: Additional file 5 — ClustalW-Neighbor Joining analysis of the genes in Rv1337 cluster. Boxed (blue) are the genes that grouped with Rv1337. Essential genes in this clade are Rv1327c, Rv1327c, Rv1331, Rv1340 and Rv1344. [file 1471-2180-10-272-S5.PDF]

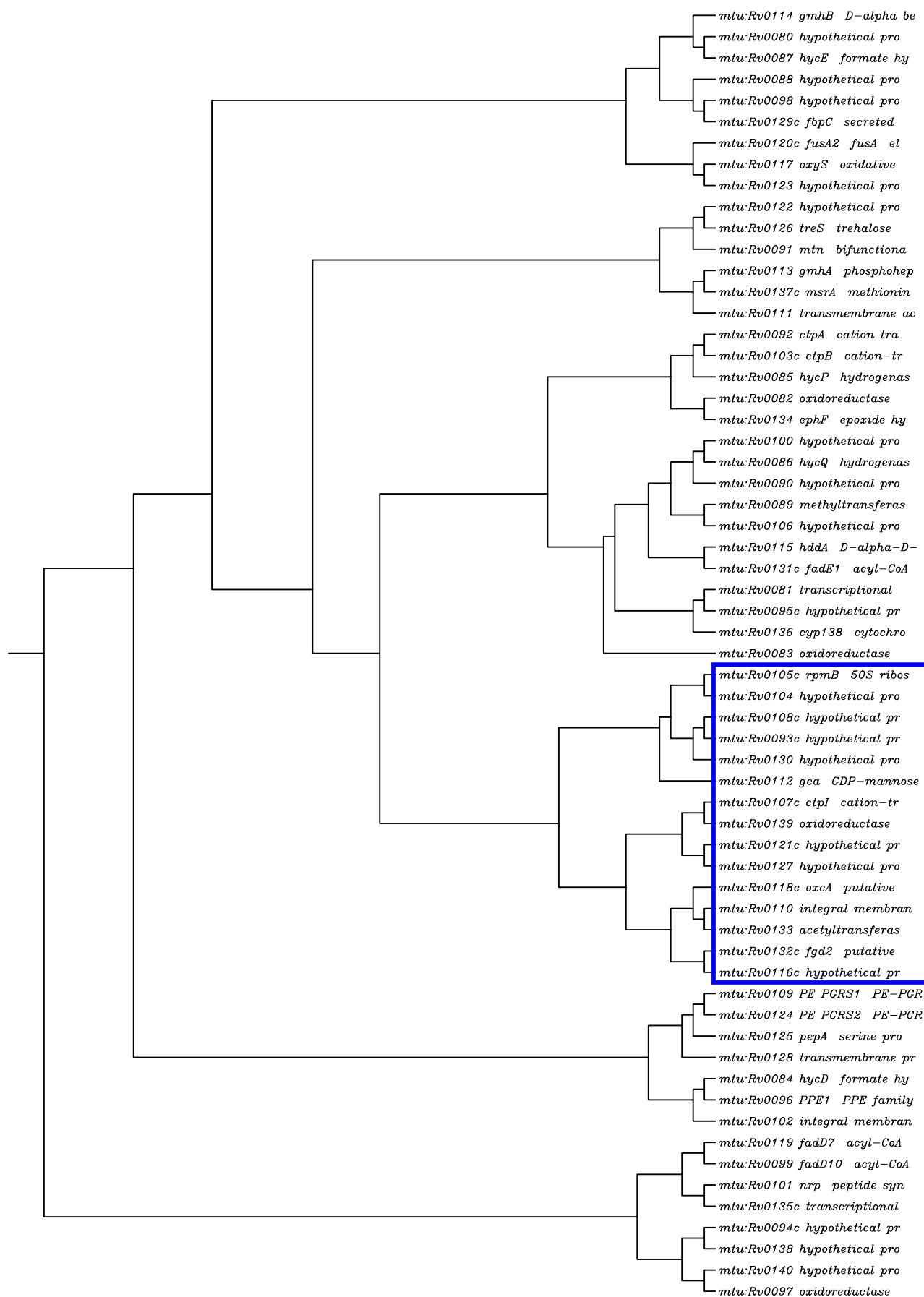

Supplement: Additional file 6 — ClustalW-Neighbor Joining analysis of the genes in Rv0110 cluster. Boxed (blue) are the essential genes in that grouped with Rv0110 (Rv0118c, Rv0127, Rv0107c, Rv0116c, Rv0121c, Rv0132c, Rv0133 and Rv0139). [file 1471-2180-10-272-S6.PDF]

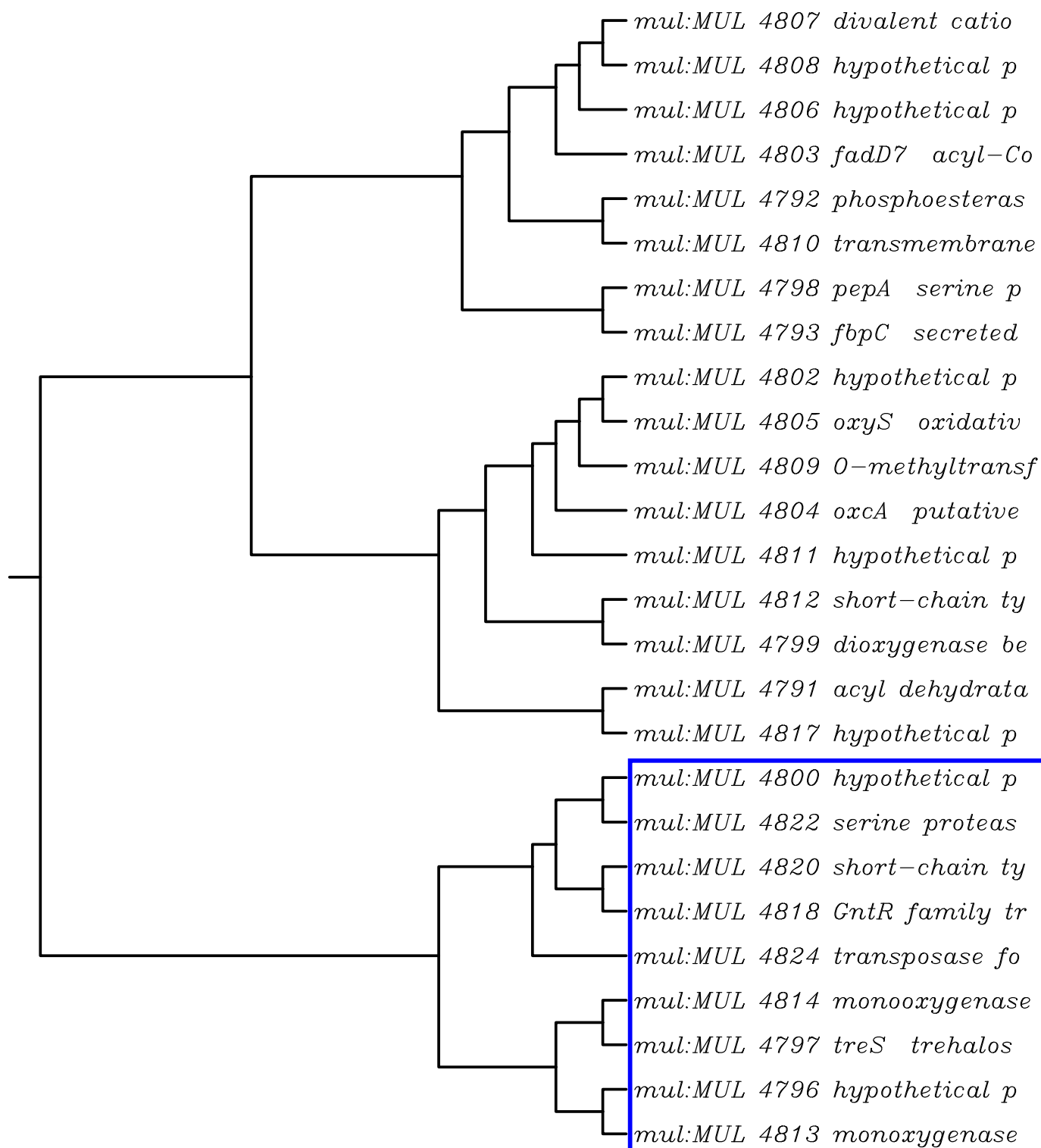

Supplement: Additional file 7 — ClustalW-Neighbor Joining analysis of the genes in MUL4822 cluster. Boxed (blue) are the genes that grouped with MUL4822. Several of the MTC orthologs in this clade are essential for the growth of M. tuberculosis in macrophages. [file 1471-2180-10-272-S7.PDF]

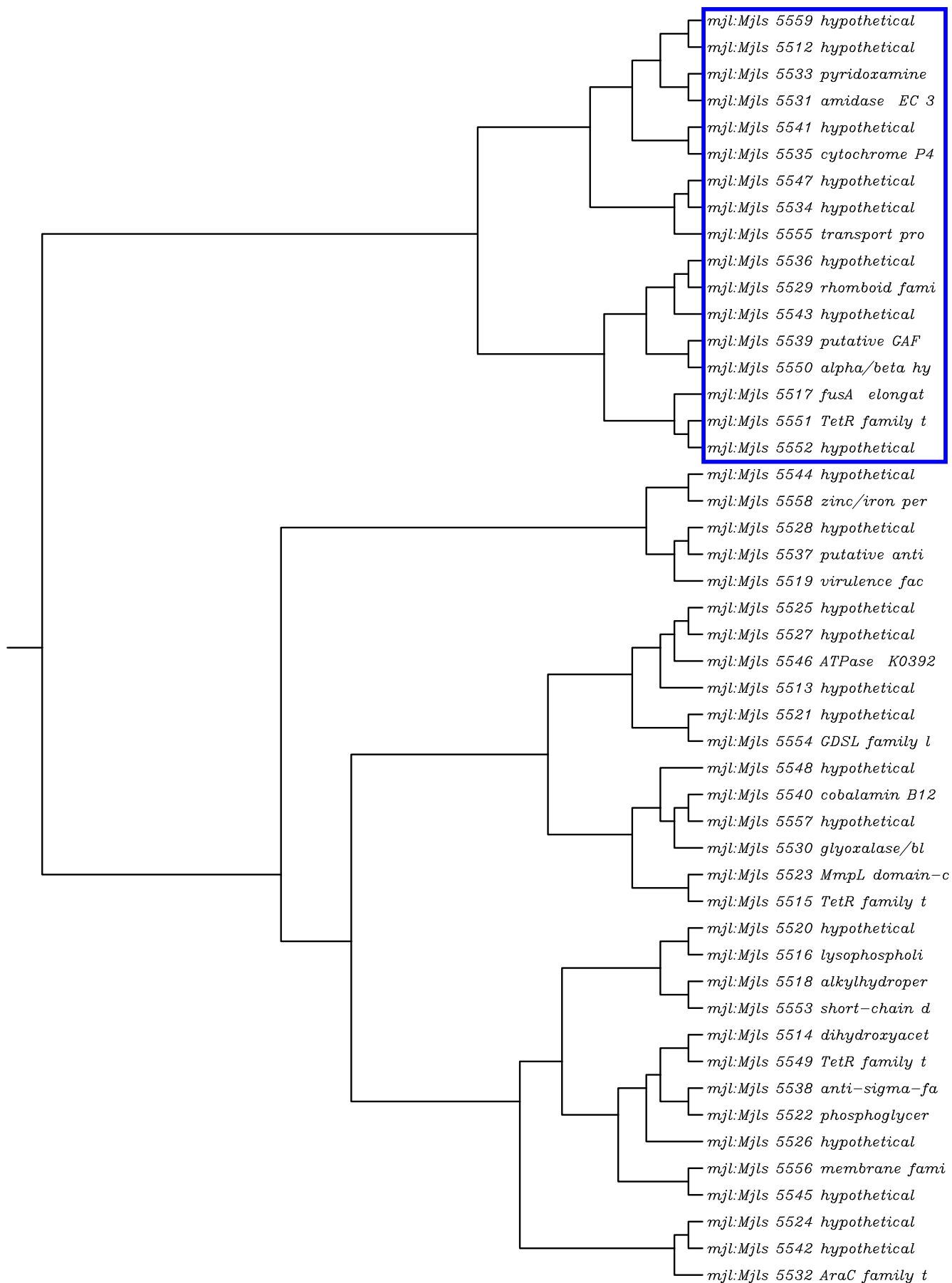

Supplement: Additional file 8 — ClustalW-Neighbor Joining analysis of the genes in Mjls5529 cluster. Boxed (blue) are the genes that grouped with Mjls5529, whose homologs are essential in M. tuberculosis. Several of the MTC orthologs in this clade are essential for the growth of M. tuberculosis in macrophages. [file 1471-2180-10-272-S8.PDF]
