# Supplementary material for: Rhomboid homologs in mycobacteria: insights from phylogeny and genomic analysis
Source: BMC Microbiol. 2010 Oct 29;10:272. doi: 10.1186/1471-2180-10-272 (PMC2989971; doi:10.1186/1471-2180-10-272)
Supplement: Additional file 9 — The essential genes in mycobacterial rhomboid gene clusters (doc). a: According to Sassetti et al [37] and Rengarajan et al [38]. 1: Essential (for optimal growth). 2: Required for growth in macrophage. 3: Mutation slows growth. [file 1471-2180-10-272-S9.DOC]

**Essential^a^ genes in mycobacterial rhomboid clusters**

| *Rv0110 gene cluster* | | |
| --- | --- | --- |
| **Name** | **Rv designation** | **Role/Category** |
| Rv0082 | Rv0082**^2^** | Oxidoreductase |
| hycP | Rv0085**^1,2^** | Hydrogenase |
| hycQ | Rv0086**^1,2^** | Hydrogenase |
| ctpA | Rv0092**^2^** | Cation transporter P-type ATPase A |
| PPE1 | Rv0096**^2^** | PPE family protein |
| Rv0102 | Rv0102**^1^** | Integral membrane protein; K07245 putative copper resistance protein D |
| ctpI | Rv0107c**^2^** | Cation-transporter ATPase I |
| Gca | Rv0112**^1,2^** | GDP-mannose 4,6-dehydratase |
| Rv0116c | Rv0116c**^2^** | Hypothetical protein |
| oxcA | Rv0118c**^1^** | Putative oxalyl-CoA decarboxylase |
| Rv0121c | Rv0121c**^2^** | Hypothetical protein |
| treS | Rv0126**^3^** | Trehalose synthase; K05343 maltose alpha-D-glucosyltransferase |
| Rv0127 | Rv0127**^1^** | Hypothetical protein |
| fgd2 | Rv0132c**^2^** | Putative F420-dependent glucose-6-phosphate dehydrogenase |
| Rv0133 | Rv0133**^2^** | Acetyltransferase |
| Rv0139 | Rv0139**^2^** | Oxidoreductase |
| *Rv1337 gene cluster* | | |
| atpC | Rv1311**^1^** | F0F1 ATP synthase subunit epsilon; K02114 F-type H+ transporting ATPase subunit epsilon |
| murA | Rv1315**^1^** | UDP-N-acetylglucosamine 1-carboxyvinyltransferase |
| glgB | Rv1326c**^1^** | Glycogen branching enzyme |
| glgE | Rv1327c**^1^** | Glucanase |
| glgP | Rv1328**^2,3^** | Glycogen phosphorylase |
| clpS | Rv1331**^2^** | ATP-dependent Clp protease adaptor protein |
| Rv1332 | Rv1332**^2^** | Transcriptional regulatory protein |
| Rv1334 | Rv1334**^2^** | Hypothetical protein |
| Rv1337 | Rv1337**^2^** | Integral membrane protein (rhomboid family) |
| Rv1339 | Rv1339**^1, 2^** | Hypothetical protein |
| Rph | Rv1340**^2^** | Ribonuclease PH |
| Rv1342c | Rv1342c**^1^** | Hypothetical protein |
| Rv1344 | Rv1344**^2^** | Acyl carrier protein |
| fadD33 | Rv1345**^2,3^** | Long-chain-fatty-acid--[acyl-carrier-protein] ligase |
| Rv1347c | Rv1347c**^1^** | Hypothetical protein |
| Rv1348 | Rv1348**^1^** | Drugs-transport transmembrane ATP-binding protein ABC transporter |
| Rv1349 | Rv1349**^1^** | Drugs-transport transmembrane ATP-binding protein ABC transporter |
| fabG | Rv1350**^1,2^** | 3-ketoacyl-(acyl-carrier-protein) reductase |
| Rv1353c | Rv1353c**^2^** | Transcriptional regulatory protein |
